# Transcriptional dynamics of maize leaves, pollens and ovules to gain insights into heat stress-related responses

**DOI:** 10.3389/fpls.2023.1117136

**Published:** 2023-02-15

**Authors:** Ashok Babadev Jagtap, Inderjit Singh Yadav, Yogesh Vikal, Umesh Preethi Praba, Navneet Kaur, Adeshpal Singh Gill, Gurmukh S. Johal

**Affiliations:** ^1^ School of Agricultural Biotechnology, Punjab Agricultural University, Ludhiana, India; ^2^ Department of Botany and Pathology, Purdue University, West Lafayette, IN, United States

**Keywords:** heat stress, maize, RNA-seq, comparative transcriptomics, differentially expressed genes (DEGs)

## Abstract

Heat stress (HS) is one of the alarming issues today due to global warming and is the foremost detrimental to crop production. Maize is one of the versatile crops grown over different agro-climatic conditions. However, it is significantly sensitive to heat stress, especially during the reproductive phase. The heat stress tolerance mechanism is yet to be elucidated at the reproductive stage. Thus, the present study focused on identifying transcriptional changes in two inbreds, LM 11 (sensitive to HS) and CML 25 (tolerant to HS), under intense heat stress at 42°C during the reproductive stage from three tissues viz. flag leaf, tassel, and ovule. Samples from each inbred were collected after 5 days of pollinations for RNA isolation. Six cDNA libraries were constructed from three separate tissues of LM 11 and CML 25 and sequenced using an Illumina HiSeq2500 platform. A total of 2,164 (1127 up-regulated and 1037 down-regulated) differentially expressed genes (DEGs) were identified with 1151, 451, and 562 DEGs in comparisons of LM 11 and CML 25, corresponding to a leaf, pollen, and ovule, respectively. Functional annotated DEGs associated with transcription factors (TFs) *viz*. AP2, MYB, WRKY, PsbP, bZIP, and NAM, heat shock proteins (HSP20, HSP70, and HSP101/ClpB), as well as genes related to photosynthesis (PsaD & PsaN), antioxidation (APX and CAT) and polyamines (Spd and Spm). KEGG pathways analyses showed that the metabolic overview pathway and secondary metabolites biosynthesis pathway, with the involvement of 264 and 146 genes, respectively, were highly enriched in response to heat stress. Notably, the expression changes of the most common HS-responsive genes were typically much more significant in CML 25, which might explain why CML 25 is more heat tolerant. Seven DEGs were common in leaf, pollen, and ovule; and involved in the polyamines biosynthesis pathway. Their exact role in maize heat stress response would warrant further studies. These results enhanced our understanding to heat stress responses in maize.

## Introduction

Diverse environmental challenges pose a severe threat and renewed concern to the world’s food security for the burgeoning human population (Tiwari and Yadav, 2019). Climate change might result in a wide variety of impacts on agricultural production (Porter, 2005). The development of genotypes with enhanced abiotic stresses is paramount to reinforce crop productivity and produce enough food to meet the demands of the predicted global population in 2050 (Gilliham et al., 2017). Drastic temperature fluctuations due to climate change frequently occur during plant growth and development (Bita and Gerats, 2013). High temperature stresses exclusively during reproductive phenophase are becoming the main concern for plant scientists under the fast-changing climatic scenario, affecting crop production and productivity worldwide (Tiwari and Yadav, 2019). Therefore, it is imperative to build up heat-resilience crop plants to cope with high temperatures due to climate change.

To gain a comprehensive understanding of the molecular mechanisms involved in heat stress (HS) tolerance, next-generation sequencing (NGS) approaches like transcriptomics or RNA-sequencing (RNA-seq) is a powerful tool for whole-genome gene expression profiling and is especially useful for studying complex gene regulatory networks (McGettigan, 2013; Frey et al., 2015). Transcriptomics has been broadly studied in several crops species like maize (Fernandes et al., 2008; Frey et al., 2015; Shi et al., 2017; Qian et al., 2019; Li and Ye, 2022; Xuhui et al., 2022), rice (Zhang et al., 2013), wheat (Qin et al., 2008; Nandha et al., 2019; Azameti et al., 2022), pepper (Li et al., 2015) and barley (Mangelsen et al., 2011). Various genes and metabolites get activated under heat stress, such as transcriptional factors, hormones, and Heat Shock Proteins (HSPs) that play a crucial role in heat stress tolerance. However, very few studies reported the comparative transcriptome analysis between heat-tolerant and heat-sensitive cultivars of crop plants- rice, maize, and pepper (Shi et al., 2017).

Maize (*Zea mays* L.), the ‘Queen of Cereals’, is the third most important cereal crop globally (Rouf Shah et al., 2016). It is one of the most versatile emerging C4 crops having high plasticity under diverse agro-climatic conditions across the globe. Forthwith, maize is grown in regions with prevailing 18-27°C habitually optimum temperatures. Nevertheless, it can also be raised at 33-38°C with optimum yield (Tiwari and Yadav, 2019). Temperatures beyond 38°C will drastically impact the heat stress on the maize crop, and consequently, the economic productivity of maize will be less (Koirala et al., 2017). More clearly, several studies have identified high temperature (heat stress) as the main threat to future maize cultivation in distinct relevant production regions like India (Gourdji et al., 2013). Heat stress during the flowering stage in maize decreases chlorophyll content, reduced membrane-thermostability, increases anthesis-silking interval (ASI), causes leaf firing and tassel blast, and reduces pollen viability and yield (Hussain et al., 2019; Inghelandt et al., 2019; Martins et al., 2019; Noor et al., 2019). The morphological and physiological effect of maize under heat stress is discussed in detail by El-Sappah et al. (2022) and Tas (2022).

It has been elucidated that transcription factors (TFs), heat shock proteins (HSPs) response pathways, response to reactive oxygen species (ROS), increasing production of antioxidant and osmoprotectants, and network of hormones participate in plant heat tolerance (Qian et al., 2019). The recent findings revealed that TFs belonging to AP2/EREBP, MYB, WRKY, bHLH, NAC, and bZIP families play a vital role in regulating heat stress-related responses at the molecular level (Li et al., 2017; Li and Ye, 2022). HSPs function as molecular chaperones and regulate the folding, localization, accumulation, and degradation of protein molecules, and induce the endoplasmic reticulum-localized unfolded protein response (ER-UPR) (Kotak et al., 2007; Hu et al., 2009; Ul haq et al., 2019; Li and Howell, 2021). Also, ascorbate peroxidase (APX) and catalase (CAT) detoxify the ROS produced during heat stress (Asthir, 2015). Moreover, several plant hormones like auxins, abscisic acid (ABA), jasmonic acid (JA), cytokinins (CKs), ethylene, gibberellin, and brassinosteroids are involved in heat stress tolerance (Li et al., 2015; Li et al., 2017).

Presently, little is known about the molecular mechanism of heat resilience in maize (Qian et al., 2019). The transcriptomic responses of maize to heat stress have been reported in a few studies and mainly focused on gene expression changes at the seedling stage (Frey et al., 2015; Li et al., 2017; Shi et al., 2017; Qian et al., 2019; Zhao et al., 2019; Li and Ye, 2022; Xuhui et al., 2022). To our best knowledge, heat stress responses in crop plants at the reproductive stage have received less attention. Moreover, transcriptomic responses in different plant parts of maize during the reproductive phase have not been elucidated yet. Therefore, it is necessary to determine the molecular mechanisms involved in heat stress in maize to understand how maize plants respond and adapt to heat stress at the reproductive stage and breed heat-resilient ready maize crops. In the present study, LM 11(heat-sensitive) and CML 25 (heat-tolerant) maize inbreds were exposed to high-temperature stress at 42°C during the flowering stage. Transcriptional dynamics among leaf, pollen, and ovule were studied to detect the differential gene expression. It has led to the identification of potential candidate genes that could be deployed for heat-resilient maize breeding.

## Materials and methods

### Plant materials and sampling

**Figure 1:**
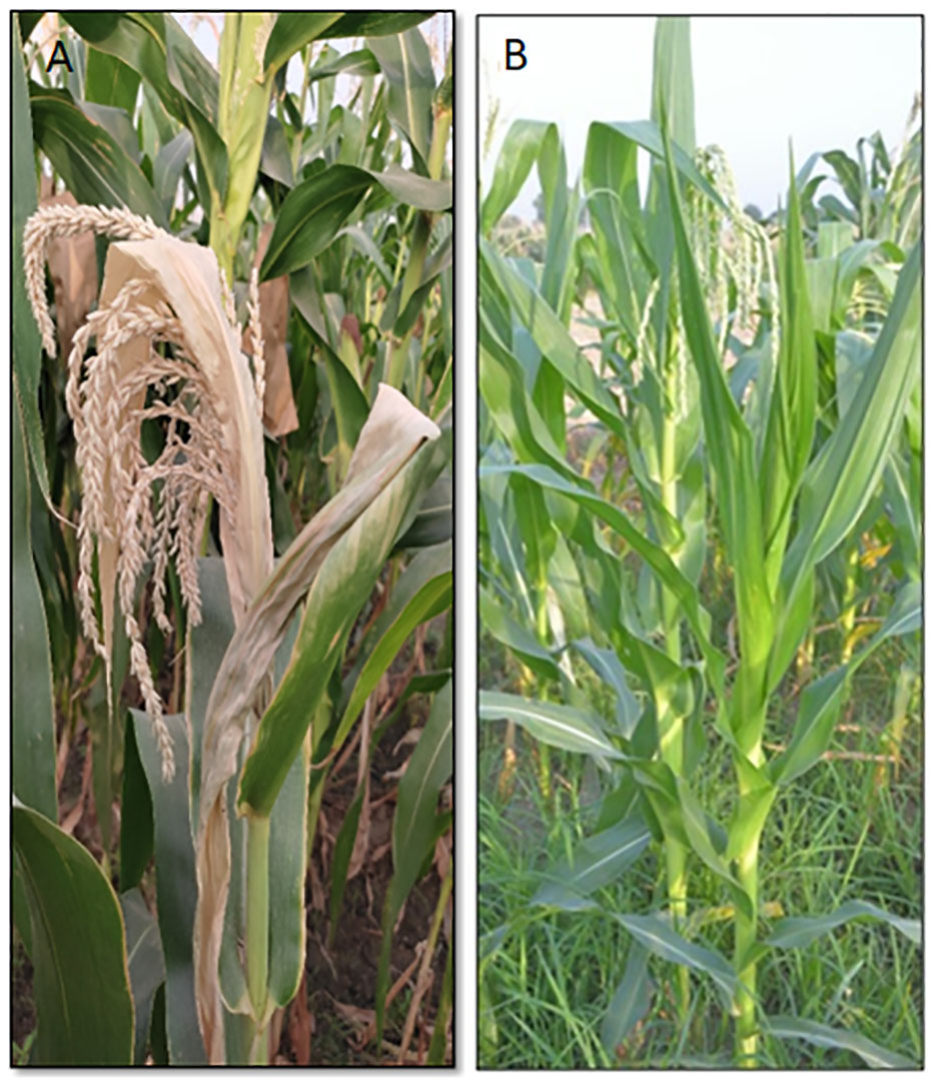
The maize inbred lines used in the present study. **(A)** LM 11 (Heat susceptible). **(B)** CML25 (Heat tolerant).

The experimental material comprised two parental maize inbred lines *viz.* LM 11, heat stress susceptible (HS), and CML 25, heat stress-tolerant (HT). The seedlings of two inbreds were raised during the second week of March in glasshouse conditions at 28°C/23°C and 16 h light (Day)/8 h dark (Night) photoperiod at the School of Agricultural Biotechnology, Punjab Agricultural University, India, during *spring* 2016. The plants were grown till they reached the reproductive stage (**Figure 1**). The reproductive phase of maize is categorized into six stages, with the emergence of tasselling and silking; followed by a blister where kernels with clear liquid get secreted and filled with milky fluid; accompanied by doughy consistency and extended kernels and milk line progression towards the kernel tip and finally, a black layer formed at the base of grains. At the reproductive growth stage, from tassel emergence to early grain-filling (lag-phase), maize plants were exposed to natural heat stress and experienced 42°C during the daytime and 35°C during the nighttime. Drought stress is confounded naturally during heat stress. To maintain the microclimate conditions with low RH (<40%) and to avoid the compound effect of drought and heat stress, regular irrigation was applied for at least two weeks during tassel emergence until one week after pollination, which increases the probability of irreversible damage due to heat stress. Both inbreds showed differential responses to heat stress for phenological attributes like top leaf firing, tassel blast, pollen viability and shedding duration, kernel number and weight, and yield (Jagtap, 2020). Three different tissue samples, *viz.* flag leaf, pollen, and ovule from the inbreds, LM 11 and CML 25, were collected in 5 replicates after five days of pollination. Tissue purity was maintained by bagging the tassel and cob. Ovules were isolated from ear florets with a silk length of ~10 cm by removing the silk and ovary wall with forceps and cutting the ovule at its base from the floret under the microscope. Each tissue was pooled (pool of five plants) for each inbred to reduce biological sampling error. A total of six samples (3 tissues x 2 inbreds) were immediately frozen in liquid nitrogen and stored at -80°C until processed for RNA isolations.

### Total RNA isolation, library construction, and Illumina sequencing

Total RNA was isolated from each tissue sample (flag leaf, pollen, and ovule) of LM 11 and CML 25 under heat stress conditions using the NucleoSpin RNA Plant kit (Macherey-Nagel, Duren, Germany) according to the manufacturer’s protocol. The RNA integrity number (RIN) and concentration were checked using an Agilent 2100 Bioanalyzer (Agilent Technologies, Inc., Santa Clara, CA, USA). The RNA (1µg) having RIN values >6 was used for further analysis. 5 μg of total RNA was used for mRNA enrichment, and complementary DNA (cDNA) library was constructed using the manufacturer’s protocol of Illumina HiSeq 2500 RNA library preparation kit (Illumina, San Diego, CA). The quality and quantity of cDNA libraries were checked using the Qubit 2.0 Fluorometer (Thermo Scientific) and Agilent 2100 Bioanalyzer (Agilent Technologies, Singapore). The libraries were then sequenced using HiSeq Illumina 2500 sequencing platform (Illumina, San Diego, CA) outsourced from the Nucleome Informatics Pvt. Ltd, Hyderabad, India. High throughput sequencing of transcriptome libraries generated an aggregate of 2.93 billion raw reads accounting for 45.19 Gb of data.

### Pre-processing and *de novo* assembly

The raw reads were processed with FastQC (Andrews, 2010) to check the quality of the sequences. Low-quality regions and adapter fragments were removed from the raw reads based on all known Illumina adapter sequences with the options 2:30:10 *via* Trimmomatic 0.36 (Bolger et al., 2014). The trimmed data were also checked for quality of sequencing before the start of further analysis using fast QC with an average PHRED score of 20. Reads below the length cut-off of 100 nucleotides were discarded. Read pairs with only one surviving read were dropped after trimming. The Q20, Q30, and GC contents were estimated to perform all downstream analyses. Transcriptome assembly was done based on the left. fq and right. fq files using Trinity v2.4.0 (Haas et al., 2013). The transcript abundance was determined at 0.1 dispersion.

### Enrichment of differentially expressed genes (DEGs)

The differential gene expression was studied using EdgeR (Robinson et al., 2010). Genes with a false discovery rate (FDR) of <0.05 and a fold change of >2 were considered as differentially expressed. The number of DEGs among and within conditions was plotted as a Venn diagram using Venny tools (http://bioinfogp.cnb.csic.es/tools/venny/) (Oliveros, 2007). Volcano plots were prepared to identify the number of transcripts regulated under heat stress conditions in different samples. The volcano center represents the fold change of zero, and either side of the center indicates the down (negative values) and up-regulation (positive values) of transcripts, respectively. Significant DEGs are represented by red and green dots with |log2 (fold change) ≥2 and FDR value less than 0.05.

#### Gene ontology and pathway enrichment analysis

DEGs and consensus sequences of isoforms were mapped to GO classifications using Blast2GO (Conesa and Götz, 2008). Gene Ontology (GO) enrichment was performed for DEGs identified in the leaf, pollen, and ovule to gain insights into their involvement in various functional annotations under heat stress conditions. Around 2,164 DEGs were subjected to GO analysis by the WEGO application (Ashburner et al., 2000). KEGG (Kyoto Encyclopedia of Genes and Genomes) pathway analysis was performed by the KOBAS2.0 packages (Xie et al., 2011). Also, the gene function annotation was accomplished by BLASTX against the databases Nr and Pfam. Pathway analysis of differentially expressed transcripts involved in specific pathways was done using MapMan version 3.6.0 RC1 with a P-value of ≤ 0.05 (Thimm et al., 2004).

### Validation of DEGs by quantitative real-time PCR (qRT-PCR)

An aliquot of total RNA isolated from heat-stressed leaf samples of LM 11 (HS) and CML 25 (HT) was used for cDNA synthesis by PrimeScript™ first strand cDNA synthesis kit (Takara, Japan) as per the manufacturer’s instructions. To validate the reliability of gene expression obtained by RNA seq, a set of six DEGs in the leaf were randomly selected for qRT-PCR. Gene-specific primers were designed using GenScript Real-time PCR (TaqMan) Primer Design tool (https://www.genscript.com/tools/real-time-pcr-tagman-primer-design-tool).

The qRT-PCR reactions were carried out in triplicate using the SYBR Premix ExTaqTM II (Takara, Japan) and run-on Light Cycler 96 Real-Time PCR system (Roche, USA). Each reaction contains 5 μl of SYBR Green Master, 0.8 μl of template cDNA, 0.4 μl of each of the primers (10 μM), and 3.4 μl of nuclease-free water with a total volume of 10 μl. The qRT-PCR profile was as follows: 2 minutes at 95°C followed by 40 cycles of 10 seconds at 95°C, 30 seconds at 60°C with fluorescent amplification signal detection, and 30 seconds at 72°C. The melting curve was obtained by PCR following the last cycle: 15 seconds at 95°C followed by constant heating between 65°C for 15 seconds and 95°C for 2 seconds. The reference gene *18S ribosomal RNA* (*rRNA*) from maize was used as an endogenous (internal) control for normalization in all the qRT-PCR analyses. Three biological replicates were performed for each sample, and data were indicated as mean ± SE (n = 3). Cycle threshold (CT) difference between the reference *18S* gene and the target gene product was used to calculate the relative expression levels of the genes using the 2^-ΔΔct^ method (Schmittgen and Livak, 2008).

## Results

### Transcript profiling under heat stress

A total of 141.03 and 152. 86 million raw reads and 125.86 and 135.61 clean reads were obtained from the cDNA sample of LM 11 and CML 25, respectively. LM 11 leaf, pollen, and ovule had 56.88, 38.71, and 45.43 million raw reads, while 52.92, 44.85, and 55.09 million raw reads from leaf, pollen, and ovule, respectively, were obtained from CML 25. The reads with adapter contamination and low base quality (≤ Q20) were removed, and high-quality (HQ) clean reads were retained. The maximum number of raw and clean base reads were obtained in the LM 11 leaf, followed by the CML 25 ovule, CML 25 Leaf, LM 11 ovule, and other samples (**Table 1**). The overall GC content ranged from 54.71 to 61.2%. A higher number of transcripts were observed in class 200–1000 bp. The principal component analysis (PCA) of the above-mentioned data (**Supplementary Figure S1**) suggests that the RNA-seq results meet the requirement of DEG identification. It is the first transcriptome library reported for heat stress in maize from three different tissues, which affects the synthesis of kernels as high temperature affects the reproductive stage of the plant (Jagtap et al., 2020).

**Table 1:** Statistics of RNA-seq data obtained from three tissues of LM 11 and CML 25 inbreds under heat stress at reproductive phase.

Inbred	Tissue	Raw reads	Clean reads	Raw bases (Gb)	Clean bases (Gb)	GC content (%)
LM 11	leaf	56883812	50600778	8.72	7.28	60.27
	pollen	38714402	34675722	5.97	4.99	56.82
	ovule	45436160	40586048	6.99	5.85	55.02
CML 25	leaf	52920330	46307576	8.12	6.63	61.20
	pollen	44852338	39999254	6.89	5.76	57.08
	ovule	55090774	49309984	8.5	7.06	54.71

### Differentially expressed genes (DEGs) under heat stress

**Figure 2:**
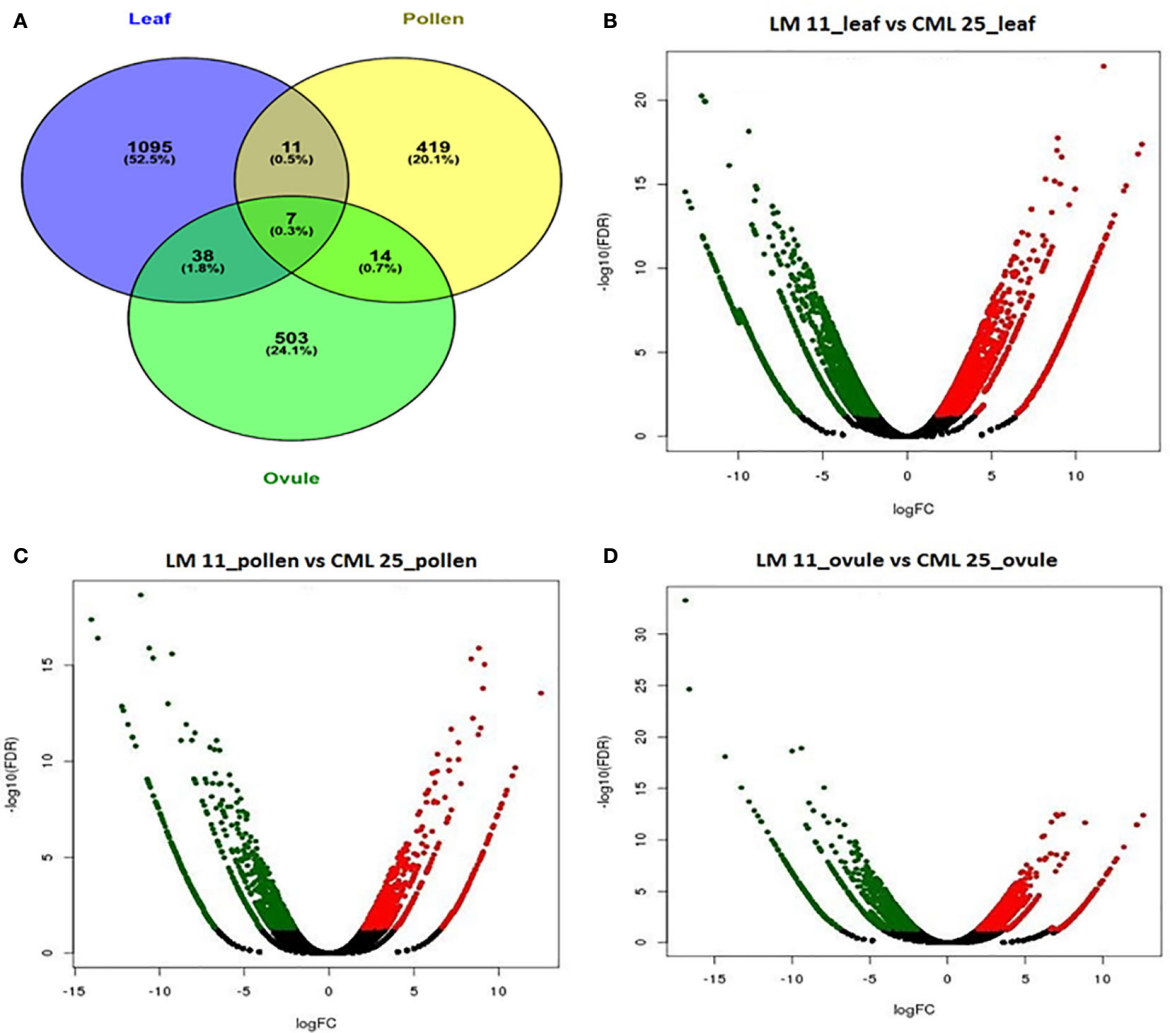
Differentially expressed genes (DEGs) involved in response to heat stress in maize at reproductive stage from three different tissues. **(A)** Venn diagram of DEGs in response to changes in leaf, pollen, and ovule. **(B)** Volcanic plot displaying DEGs in leaf of LM11 (S) versus CML 25 (T). **(C)** Volcanic plot displaying DEGs in pollen of LM 11 (S) versus CML 25 (T). **(D)** Volcanic plot displaying DEGs in ovule of LM 11 (S) versus CML 25 (T). Red dots represent up-regulated DEGs; green dots represent down-regulated DEGs. The x-axis values correspond to the log_2_ (fold change) value; the y-axis corresponds to the mean expression value of the −log10 (p-value) between LM 11 and CML 25. The Volcano plots for differentially expressed transcripts represent FDR ≤ 0.05 and |log_2_ (fold change) ≥2.

A total of 2,164 transcripts were differentially expressed between LM 11 and CML 25. 1151, 451, and 562 DEGs were specific in LM 11 versus CML 25 leaf, pollen, and ovule, respectively. A total of 1,095 (52.5%), 419 (20.1%), and 503 (24.1%) DEGs were uniquely expressed in LM 11 leaf versus CML 25 leaf, LM 11 pollen versus CML 25 pollen and LM 11 ovule versus CML 25 ovule, respectively (**Figure 2A**). Eleven (0.5%) and thirty-eight (1.8%) DEGs were common between leaves with pollen and ovule, respectively. 14 (0.7%) and 7 (0.3%) DEGs were similar between pollen and ovule and between all three samples, respectively (**Figure 2A**). Likewise, in LM11_leaf versus CML25_leaf, 578 DEGs were up-regulated, and 573 DEGs were down-regulated. Similarly, in LM11_pollen versus CML25_pollen, 231 DEGs were up-regulated, and 220 DEGs were down-regulated. While in LM11_ovule versus CML25_ovule, 318 and 244 DEGs were up-regulated and down-regulated, respectively (**Figures 2B–D**). In addition, a heat map of the overall expression pattern of the DEGs revealed that many unique DEGs were highly expressed in CML 25 leaf and LM 11 leaf samples compared to pollen and ovule samples (**Supplementary Figure S2**).

The highest up-regulated and down-regulated DEGs in the leaf, pollen, and ovule are summarized in **Tables 2**, **3**. Nine pollen DEGs showed high expression encoding for a beta-expansion protein, endoglucanase, serine/threonine protein kinase, pectin esterase, beta-amylase, and acid phosphatase family proteins. One DEG with the highest expression level in the leaf is involved in cytochrome b6-f complex iron-sulfur subunit (**Table 2**). Likewise, out of the top 10 down-regulated DEGs, six were found in pollen and four in the leaf. The down-regulated DEGs in pollen encode endoglucanase, pectin esterase inhibitor, beta-amylase, glucan endo-1,3 beta-glucosidase, and pollen-specificity family proteins. Four highly down-regulated DEGs in the leaf are involved in dehydrin, calcium-dependent protein kinase, glycerate dehydrogenase, and eukaryotic translation initiation factor 3 subunit proteins (**Table 3**).

**Table 2:** Top 10 up-regulated DEGs in leaf, pollen, and ovule in comparisons of LM 11 (S) and CML 25 (T) inbreds under heat stress conditions.

Transcript Id	Tissue	Log_2_ Fold Change	P-value	FDR	Transcript description	Total GO term	GO IDs
comp84863_c0_seq1	leaf	9.66	5.14E-24	8.42E-20	cytochrome b6-f complex iron-sulfur subunit	8	F:GO:0009496; C:GO:0009535; P:GO:0015979; C:GO:0016021; P:GO:0022900; F:GO:0045158; F:GO:0046872; F:GO:0051537
comp80999_c0_seq5	pollen	9.43	5.78E-23	9.04E-20	beta-expansin 1a precursor	5	C:GO:0005576; C:GO:0005618; C:GO:0016020; P:GO:0019953; P:GO:0071555
comp74578_c0_seq2	pollen	9.20	1.15E-22	1.61E-19	endoglucanase 8	4	C:GO:0005576; F:GO:0008810; P:GO:0030245; P:GO:0071555
comp80312_c0_seq1	pollen	9.03	9.48E-22	7.85E-19	serine/threonine-protein kinase At5g01020-like	5	F:GO:0004675; F:GO:0005524; C:GO:0005886; P:GO:0006468; P:GO:0007178
comp73316_c0_seq1	pollen	9.00	4.97E-22	5.00E-19	pollen allergen Phl p 11	1	C:GO:0005615
comp66485_c0_seq2	pollen	8.91	3.76E-22	4.08E-19	Pectinesterase family protein, expressed	5	C:GO:0005618; F:GO:0030599; P:GO:0042545; F:GO:0045330; P:GO:0045490
comp72137_c0_seq1	pollen	8.90	7.23E-22	6.37E-19	pectinesterase inhibitor domain containing protein	4	F:GO:0030599; P:GO:0043086; F:GO:0046910; C:GO:0071944
comp80939_c0_seq2	pollen	8.59	4.80E-21	3.38E-18	beta-amylase	4	P:GO:0000272; C:GO:0009507; F:GO:0016161; F:GO:0102229
comp78238_c0_seq1	pollen	8.45	3.51E-20	2.35E-17	acid phosphatase 1 precursor	2	F:GO:0003993; P:GO:0016311
comp83327_c1_seq5	pollen	8.34	2.35E-19	1.33E-16	predicted protein	1	F:GO:0005509

**Table 3:** Top 10 down-regulated DEGs in leaf, pollen, and ovule in comparisons of LM 11 and CML 25 inbreds under heat stress conditions.

Transcript Id	Tissue	Log_2_ Fold Change	P-value	FDR	Transcript description	Total GO term	GO IDs
comp74586_c0_seq2	pollen	-10.53	6.32E-26	2.22E-22	endoglucanase 8	4	C:GO:0005576; F:GO:0008810; P:GO:0030245; P:GO:0071555
comp72137_c0_seq3	pollen	-10.33	1.09E-25	3.07E-22	pectinesterase inhibitor domain containing protein	4	F:GO:0030599; P:GO:0043086; F:GO:0046910; C:GO:0071944
comp77346_c1_seq10	leaf	-9.95	1.64E-24	5.37E-20	dehydrin COR410	3	P:GO:0006950; P:GO:0009415; F:GO:0046872
comp80939_c4_seq2	pollen	-9.62	6.87E-24	1.38E-20	beta-amylase 1, chloroplastic-like	4	P:GO:0000272; C:GO:0009507; F:GO:0016161; F:GO:0102229
comp63912_c0_seq2	leaf	-8.38	6.50E-20	3.55E-16	calcium-dependent protein kinase, isoform AK1	9	F:GO:0004683; F:GO:0005516; F:GO:0005524; C:GO:0005634; C:GO:0005737; F:GO:0009931; P:GO:0018105; P:GO:0035556; P:GO:0046777
comp79197_c3_seq1	pollen	-7.77	3.20E-18	1.45E-15	glucan endo-1,3-beta-glucosidase 8-like	5	F:GO:0004553; P:GO:0005975; C:GO:0016021; F:GO:0030247; C:GO:0046658
comp78238_c0_seq3	pollen	-7.75	3.78E-18	1.66E-15	acid phosphatase 1 precursor	2	F:GO:0003993; P:GO:0016311
comp72119_c1_seq2	leaf	-7.37	5.24E-17	1.15E-13	glycerate dehydrogenase	5	C:GO:0005829; F:GO:0016618; F:GO:0030267; F:GO:0051287; P:GO:0055114
comp84091_c0_seq1	leaf	-7.34	4.67E-17	1.09E-13	eukaryotic translation initiation factor 3 subunit I-like	5	P:GO:0001732; F:GO:0003743; C:GO:0005852; C:GO:0016282; C:GO:0033290
comp63821_c1_seq1	pollen	-7.32	4.22E-17	1.49E-14	pollen-specific protein C13-like	1	C:GO:0005615

### Gene ontology (GO) classification of DEGs

**Figure 3:**
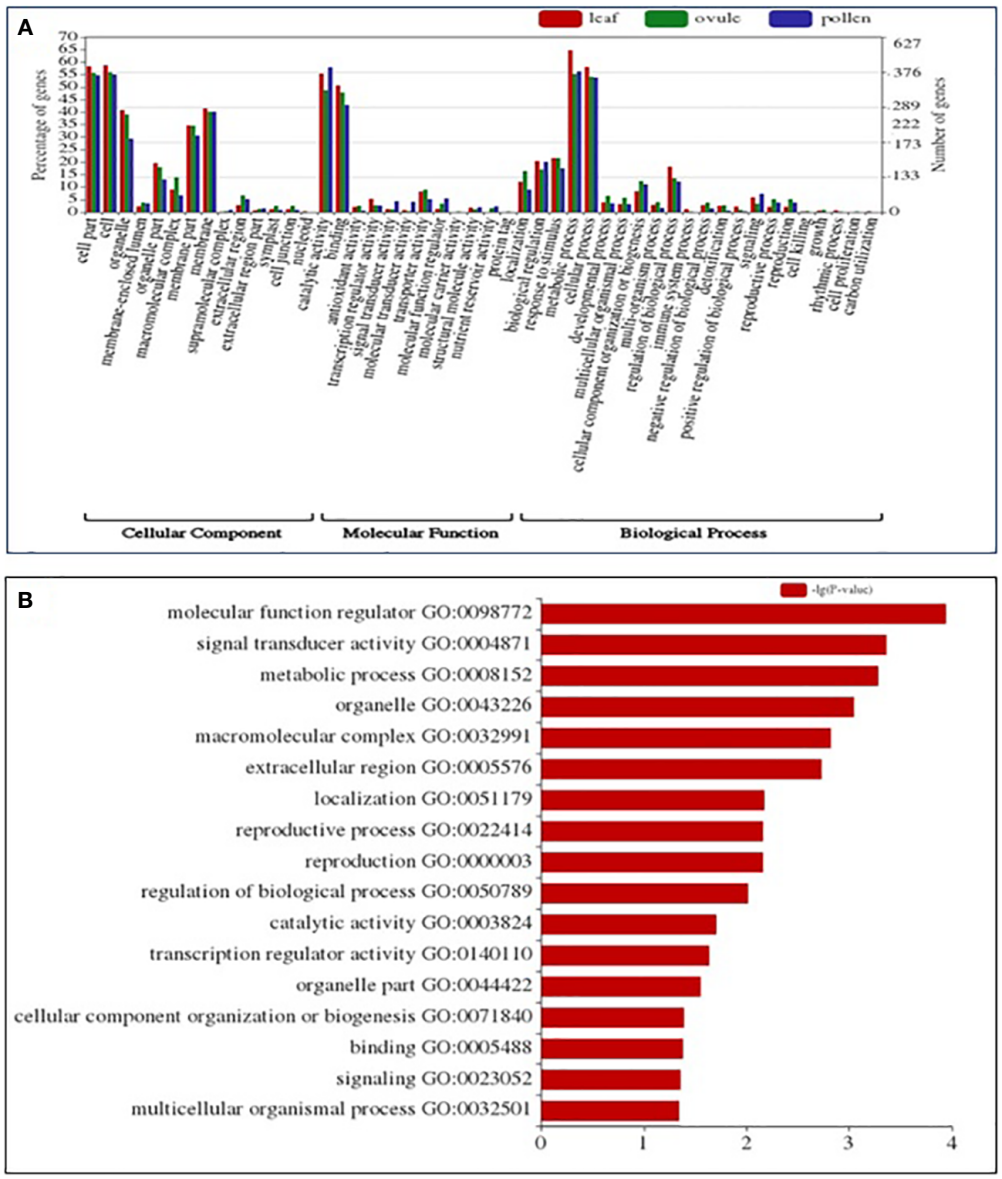
GO and KEGG enrichment analysis of DEGs identified in the comparison of leaf, pollen, and ovule of LM 11 (S) and CML 25 (T) under heat stress. **(A)** GO analysis categorized DEGs into Cellular Component (CC), Molecular Function (MF), and Biological Process (BP). The right Y-axis indicates the number of genes in GO categories. The left Y-axis indicates the percentage of a specific category of genes in that main category. The X -axis defines GO terms. **(B)** Abundant KEGG enrichment assigned to DEGs in leaf, pollen, and ovule, collectively under heat stress.

A total of 238 enriched GO terms were assigned to DEGs based on stringent p-value (0.001) and q-value (0.001) (**Figure 3A**) in LM 11 versus CML 25 leaf under heat stress conditions. These GO terms were further categorized into 132 (55.47%) biological process (BP), followed by 60 (25.21%) cellular component (CC) and 46 (19.32%) molecular function (MF) (**Supplementary Table S1**). While in LM 11 versus CML 25, pollen GO terms were categorized into 89 (48.90%) BP, followed by 47 (25.82%) CC and 46 (25.27%) MF (**Supplementary Table S2**). Correspondingly, a total of 216 enriched GO terms in ovule were assigned to DEGs and were classified into 114 (52.77%) BP, followed by 53 (24.53%) CC and 49 (22.68%) MF (**Supplementary Table S3**).

Exclusively in leaf, pollen, and ovule under the BP category, the term “metabolic process” (GO:0008152) and “cellular process” (GO:0009987) were commonly highly enriched components. The top two enriched terms under CC were “cell” (GO:0005623) and “cell part” (GO:0044464). Catalytic (GO:0003824) and binding activity (GO:0005488) were highly heightened under the MF category. Collectively, molecular function regulator (GO:0098772), signal transducer activity (GO:0004871), and metabolic process (GO:0008152) were highly enriched in leaf, pollen, and ovule. Exclusively, the metabolic process (GO:0008152) was prevalent in leaf, pollen, and ovule (**Figure 3B**).

### Metabolic pathways enrichment analysis

**Figure 4:**
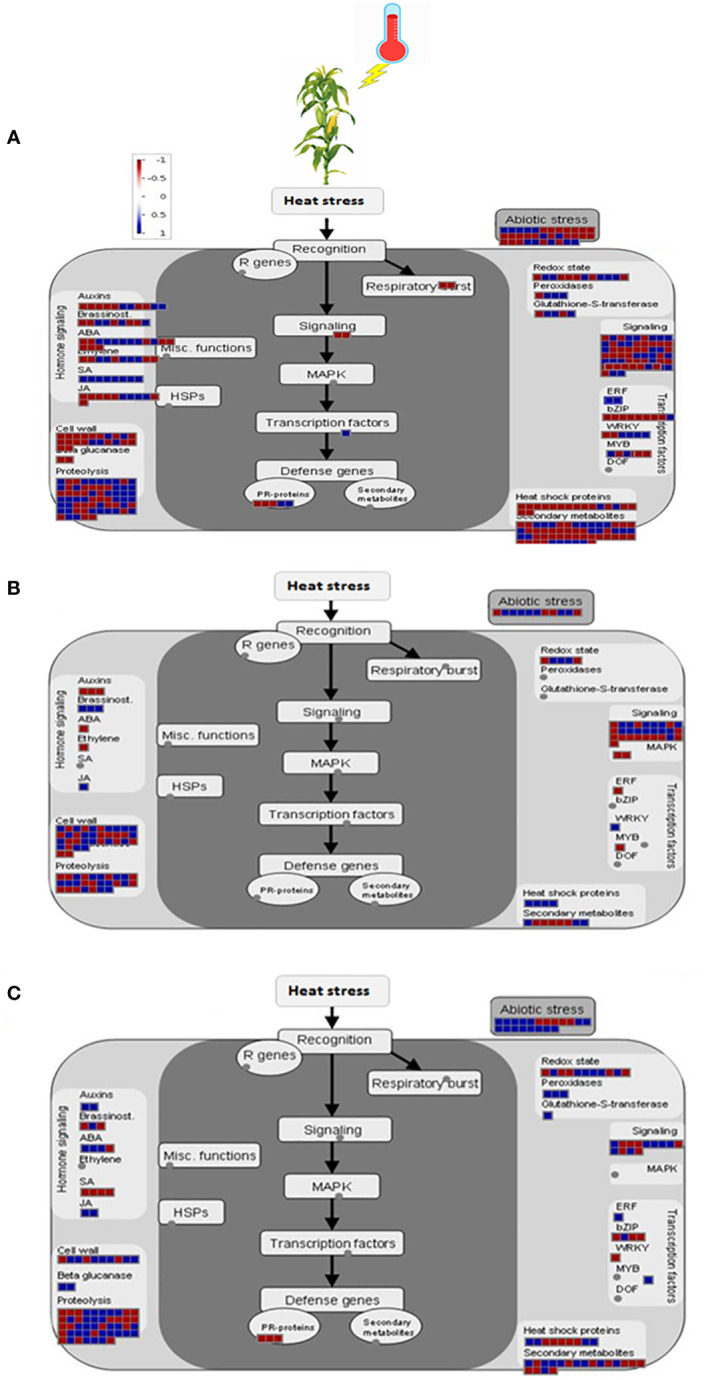
MapMan display for heat responsive genes in leaf **(A)**, pollen **(B)**, and ovule **(C)**. Biological functions of overall heat responsive genes at FDR < 0.05 and |log_2_ (fold change)| ≥ 2 under heat stress conditions. Colors represent log_2_ fold changes higher (blue) for up-regulated and lower (red) for down-regulated than 0.

KEGG pathway analysis of 2164 DEGs from all the tissues revealed that 264 and 146 genes were involved in the metabolic overview pathway and biosynthesis of secondary metabolites, respectively. Apart from them, carbon metabolism, starch and sucrose metabolism, biosynthesis of amino acids, protein processing in the endoplasmic reticulum, plant hormone signal transduction, plant-pathogen interactions, the amino sugar, and nucleotide sugar metabolism, and arginine & proline metabolism were significantly involved in response to heat stress (**Table 4**). MapMan displayed the involvement of nearly all the pathways concerning DEGs in the leaf. Among the key pathways, proteolysis had the highest number of BINs (65), followed by signaling (57), secondary metabolite (55), and abiotic stresses (34), which represented the functional categories to which genes were assigned (**Figure 4A**). Similarly, in pollen, the cell wall had the highest number of BINs (34), followed by signaling (28), proteolysis (27), and abiotic stresses (11) (**Figure 4B**). Furthermore, in the ovule, proteolysis had the highest number of BINs (47), followed by abiotic stresses (20), secondary metabolite (19), redox state (11), and cell wall (**Figure 4C**).

**Table 4:** Top twenty KEGG pathway enrichment of the DEGs of leaf, pollen and ovule under heat stress conditions.

Term	ID	Input number	Background number	P-Value	Corrected P-Value
Metabolic pathways	zma01100	264	2609	4.68E-39	5.20E-37
Biosynthesis of secondary metabolites	zma01110	146	1481	1.04E-20	5.75E-19
Carbon metabolism	zma01200	33	372	6.61E-05	0.000458578
Starch and sucrose metabolism	zma00500	31	251	2.56E-07	7.10E-06
Biosynthesis of amino acids	zma01230	28	371	0.002236704	0.008866932
Protein processing in endoplasmic reticulum	zma04141	26	316	0.001038062	0.004608996
Plant hormone signal transduction	zma04075	26	355	0.004522581	0.015687704
Plant-pathogen interaction	zma04626	25	209	5.90E-06	7.27E-05
Amino sugar and nucleotide sugar metabolism	zma00520	24	186	2.80E-06	4.10E-05
Arginine and proline metabolism	zma00330	22	78	3.56E-11	1.32E-09
Oxidative phosphorylation	zma00190	22	170	6.85E-06	7.60E-05
Ribosome	zma03010	22	504	0.399824335	0.558433653
Phenylpropanoid biosynthesis	zma00940	21	242	0.001661749	0.006831636
Glycolysis/Gluconeogenesis	zma00010	20	193	0.000282769	0.001426698
Spliceosome	zma03040	19	269	0.018878607	0.053731419
Glutathione metabolism	zma00480	18	139	4.50E-05	0.000393884
Endocytosis	zma04144	18	270	0.0347085	0.087560079
Glyoxylate and dicarboxylate metabolism	zma00630	15	110	0.00011265	0.000735541
Cysteine and methionine metabolism	zma00270	15	137	0.000936044	0.004329204
Pentose and glucuronate interconversions	zma00040	14	76	1.01E-05	0.000102308

### Alterations in the expression of transcription factors (TFs)

**Figure 5:**
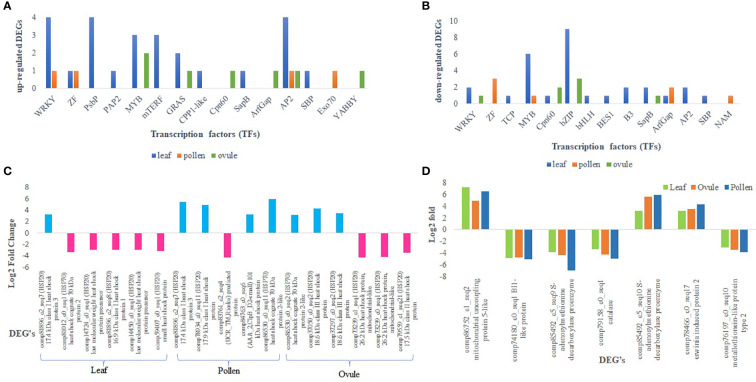
Differential expression of representative genes involved in molecular function in response to heat stress. **(A)** Transcription factors those are commonly up-regulated in leaf and ovule, and leaf and pollen. **(B)** Transcription factors those are commonly down-regulated in leaf and ovule, and leaf and pollen. **(C)** Relative expression of heat shock proteins (HSPs) in leaf, pollen, and ovule. The blue color indicates the DEGs are up-regulated and the pink color indicates DEGs are down-regulated. **(D)** Graphical representation of commonly expressed DEGs in leaf, pollen and ovule that are involved in the polyamine biosynthesis pathway.

A total of 24 classes of TFs were identified from the 55 differentially expressed transcripts of the leaf. Most TFs were attributed to MYB (Myb DNA-binding, Myb_CC_LHEQLE, Myb_DNA-bind_4, Myb_DNA-bind_6, and bZIP followed by WRKY, AP2, and PsbP. Most of the transcripts for Apetala 2 (AP2) were up-regulated in the leaf, and among all, one transcript was highly expressed with fold change of 5.22 (up-regulated) followed by WRKY (4.74) (**Figure 5A**). A total of four transcripts for PsBP were up-regulated. Most of the MYB transcripts and nine transcripts for bZIP were down-regulated (**Supplementary Table S4**). Similarly, eight classes of TF families *viz.* NAM, WRKY, zf-C2H2_6, ArfGap-C2, Myb_DNA-bind_6, zf-C3HC4_3, Exo70, and AP2 were attributed in comparisons of LM 11 versus CML 25 pollen. All differentially expressed TFs in pollen were down-regulated except WRKY, Exo70, and AP2 (**Supplementary Table S5**). Likewise, 11 classes of TF families *viz.* Asp, SapB_2, AP2, DELLA, GRAS, YABBY, bZIP, Cpn60_TCP1, WRKY, Myb_DNA-binding, and Arf were ascribed comparative to LM 11 versus CML 25 ovule. AP2, DELLA, GRAS, YABBY, Myb_DNA-binding, and Arf were up-regulated (fold changes ranging from 3.37 to 4.46), whereas, Asp, SapB_2, bZIP_1, and WRKY were down-regulated (fold changes varying from -3.31 to -5.44) (**Figure 5B**; **Supplementary Table S6**).

### Induction of metabolic processes genes

A total of 21 and 34 DEGs were up-regulated and down-regulated, respectively, in comparisons of LM 11 versus CML 25 leaf. The up-regulated DEGs are involved in sugar transmembrane transporter activity, response to reactive oxygen species (ROS), abscisic acid (ABA), oxidative stress, photosynthesis (photosystem I and chloroplast thylakoid membrane), and cytochrome-c peroxidase activity (**Supplementary Table S7**). The down-regulated DEGs have roles in the oxidation-reduction process, hydrolase and glyoxylate reductase (NADP) activity, transmembrane transport, and response to light stimulus (**Supplementary Table S8**). Twenty-three and 19 DEGs were up-regulated and down-regulated in comparisons of LM 11 versus CML 25 pollen. Up-regulated DEGs are associated with the catabolic process, cellular response to nitrogen and phosphate starvation, mitochondrial functions, and cellular response to oxidative stress (ROS) (**Supplementary Table S9**). One of the up-regulated DEGs (comp86232_c0_seq4) regulates inflorescence development, photoperiodism, and flowering. Pollen-specific NTP303 precursor protein (comp87307_c0_seq7) is involved in the oxidation-reduction process. Down-regulated DEGs belong to the zinc finger domain family protein, beta-glucosidase, alkaline/neutral invertase, vacuolar membrane, and microtubule-associated protein (**Supplementary Table S10**). One of the down-regulated DEG (comp70405_c0_seq2) was the ZIM motif family protein involved in flower development.

A total of 50 and 45 DEGs were up-regulated and down-regulated, respectively, in comparisons of LM 11 versus CML 25 ovule. Among the up-regulated transcripts, comp81007_c0_seq9, comp67757_c0_seq2, comp44452_c0_seq1, comp61084_c0_seq2, and comp13987_c0_seq1 were ovule specific in expressions as well as related to abiotic stresses. One of the DEG (comp81007_c0_seq9) belongs to lipoxygenase involved in the seed germination processes. Also, corresponding four DEGs *viz.*, comp67757_c0_seq2, comp44452_c0_seq1, comp61084_c0_seq2, and comp13987_c0_seq are related to oleosin, dehydrin, membrane protein At3g27390, and putative late embryogenesis abundant protein, respectively. These four up-regulated DEGs are involved in lipid storage, response to water deprivation, vegetative to the reproductive phase transition of the meristem, and positive regulation of response to water deprivation (**Supplementary Table S11**). Two down-regulated transcripts, comp86012_c3_seq2 and comp83060_c3_seq3, are associated with glyceraldehyde-3-phosphate dehydrogenase (NAD+) (phosphorylating) activity. Another DEG, comp86139_c1_seq7, related to beta-expansin (EXPB7), has roles in the sexual reproduction process. Similarly, DEG comp83923_c2_seq9 was histone H4 specific and is regulated in response to water stress (**Supplementary Table S12**).

### Identification of expressed HSPs

A total of fourteen, seven, and four DEGs related to HSPs were identified in leaf, pollen, and ovule, respectively. Six DEGs were highly expressed in the leaf, of which only one was up-regulated (HSP20), and five were down-regulated. In pollen, three HSP genes were up-regulated, two encoding for HSP20 and the other encoding 101 kDa heat shock protein (Hsp101/ClpB). One down-regulated DEG in pollen related to Rieske Iron-Sulfur Protein was associated with HSP70 (**Figure 5C**). Likewise, four genes for HSP20 (2 genes) and HSP70 (2 genes) were up-regulated, whereas three genes encoding for HSP20 were down-regulated in the ovule.

### Hormone biosynthesis and signal transduction-related genes

A total of 12 and 20 DEGs related to hormone biosynthesis were up-regulated and down-regulated, respectively, in comparisons of LM 11 versus CML 25 leaf. The highest up-regulated DEGs are dehydrin COR410, followed by terpene synthase 7, pyrophosphate synthase, ZIM motif family protein, and catalase. These are actively involved in the cellular response to a salicylic acid stimulus, nitric oxide and abscisic acid (ABA), regulation of jasmonic acid (JA) mediated signaling pathway and response to auxin. In addition, several other up-regulated genes belong to DNA binding, transcription factor activity, polyprenol biosynthetic process, terpene synthase activity, and response to water deprivation (**Supplementary Table S13**). Likewise, down-regulated DEGs in the leaf are related to jasmonic acid (JA), abscisic acid (ABA) synthesis, auxin-activated signaling pathway, response to water deprivation, calcium-mediated signaling, tricarboxylic acid cycle (TCA), and salicylic acid-mediated signaling pathways. Three calcium-dependent protein kinases were up-regulated in LM 11 compared to CML 25 pollen and are involved in DNA binding transcription factor activity, calmodulin-dependent protein kinase activity, calcium ion binding, calmodulin binding, ATP binding, abscisic acid-activated signaling pathway (**Supplementary Table S14**). One DEG encodes for late embryogenesis abundant protein that was highly up-regulated (fold change; 8.26) and involved in stress-related responses. Furthermore, seven DEGs were down-regulated in LM 11 pollen associated with zeaxanthin epoxidase, probable indole-3-acetic acid (IAA)-amido synthetase GH3.8, calcium-dependent protein kinase 34-like, ZIM motif family protein, gibberellin 2-oxidase, phospho-2-dihydro-3-deoxyheptonate aldolase, and HlyIII predicted protein (**Supplementary Table S14**). Similarly, 19 up-regulated DEGs in the ovule of LM 11 versus CML 25 are associated with seed-specific expressions, and one down-regulated DEG belongs to catalase and is related to the signaling pathway (**Supplementary Table S15**).

### Differential expression of polyamines biosynthesis pathway related genes

Seven polyamines biosynthesis pathway-related transcripts were differentially expressed in leaf, pollen, and ovule. Three of the genes were up-regulated in all three tissues that have roles in mitochondrial transport, adenosylmethionine decarboxylase activity, and spermine biosynthetic process. Four down-regulated genes are involved in cell-organelle-related responses under heat stress and metal ion binding (**Figure 5D**).

### qRT-PCR validation of DEGs

**Figure 6:**
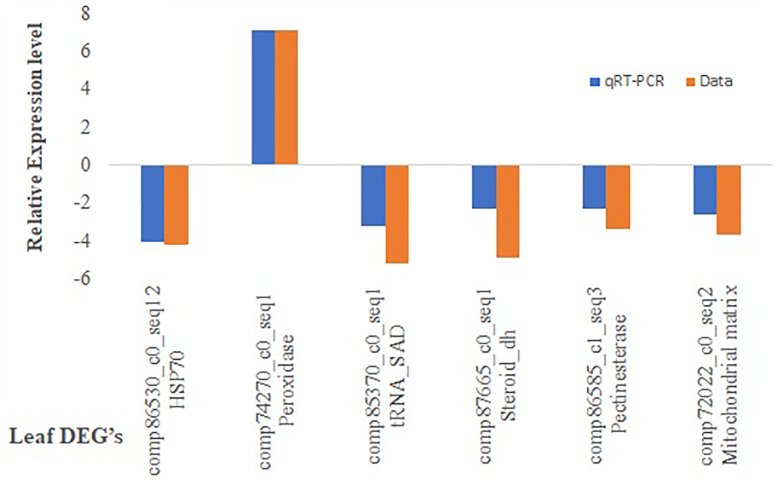
Quantitative real-time PCR (qRT-PCR) validation of DEGs characterized by RNA-seq data.

The RNAseq results were validated by selecting six genes randomly and primers were designed (**Supplementary Table S16**). The ratio of comparative expression level found between LM 11 and CML 25 was calculated and the log2 fold changes were compared with the result of RNA-seq data (**Figure 6**). The qRT-PCR data showed a significant correlation (R2 = 0.7961) with RNA-seq data, which supported the authenticity of expression patterns revealed by RNA-seq (**Supplementary Table S17**). 

## Discussion

### Transcriptome analysis

Heat stress (HS) due to an increase in global mean temperature as a result of climate change can remarkably suppress plant growth and development. It has been reported as one of the most critical causes of yield reduction and dry matter production in many crops, including maize (Lobell et al., 2008; Hasanuzzaman et al., 2013; Qian et al., 2019). It is negatively associated with anthesis, silking, and grain-filling reproductive stages (Noor et al., 2019; Longmei et al., 2021). LM 11 had a higher canopy temperature (40.13°C) than CML 25 (38.38°C) as the heat susceptible have high canopy temperature as observed in wheat and cotton genotypes (Carmo-Silva et al., 2012). The reproductive stage in plants is sensitive to heat stress, and pollen viability is directly affected by heat stress. Reduced pollen viability was observed in LM 11 (40%) under heat stress compared to CML 25 (84.04%). The impact of heat stress was more significant in LM 11 *per se* to kernel number per ear (Jagtap, 2020). Thus, there is a need to dissect physiological and molecular mechanisms underlying heat stress responses and adaptation at the reproductive stage in maize. In the past decade, the RNA-seq approach has been widely used for revealing molecular mechanisms of heat stress responses at the seedling stage (Frey et al., 2015; Shi et al., 2017; Kim et al., 2019; Li and Ye, 2022; Xuhui et al., 2022).

In the present study, comparative transcriptomics was performed for leaf, pollen, and an ovule of heat-tolerant inbred CML 25, and susceptible inbred LM 11 was analyzed under prolonged heat stress at 42°C. A total of 1127 up-regulated and 1037 down-regulated DEGs were identified in response to heat stress. The higher number of up-regulated genes indicates the cumulative activation of defense responses upon heat stress. Similarly, in heat transcriptomic studies conducted in wheat, rice, and maize, the number of induced genes was 3-6 times more than the number of repressed genes (Fernandes et al., 2008; Qin et al., 2008; Zhang et al., 2013). In the present study, leaf tissue under heat stress showed maximum DEGs (1151) followed by ovule (562) and pollen (451). The higher number of DEGs in the flag leaf tissue may be attributed to its higher metabolic activity under heat stress, as it is responsible for assimilating synthesis and translocation to the developing organs (Li et al., 2017; Shi et al., 2017; Qian et al., 2019).

In our study, the most enriched GO are biological processes comprising metabolic processes, cellular processes, and regulation. Among the molecular function category, catalytic activity and binding were mainly dominant. Also, Wang et al. (2016) categorized the enriched term “metabolic process” as a top GO term in comparative transcriptomics of Chinese cabbage to reveal the heat-responsive genes. Rahmati Ishka et al. (2018) showed that under the biological process, more than two-thirds of the over-represented genes belong to just two categories metabolic and cellular processes. It can be envisaged that crosstalk might exist among different pathways involved in various abiotic stresses (Singh et al., 2019). Thus, transcriptional reprogramming might have a role in heat stress tolerance.

### Transcription factors triggers regulatory networks

Transcription factors play fundamental roles in biotic and abiotic stresses and are considered frontline defenders. Other studies suggested that most of the identified DEGs under heat stress in crop plants encode members of the ERF, MYB, bZIP, bHLH, WRKY, NAC, and MYB-related TF families (Wang et al., 2016; Li et al., 2017; Zhao et al., 2017; Qian et al., 2019; Li and Ye, 2022) (**Table 5**). In the present study, we found several TFs expressed under heat stress in both cultivars like MYB, bZIP, WRKY, AP2, NAM, DELLA, etc., and might have a role in overcoming the heat stress in CML 25. A fraction of TFs, including 9 MYB and bHLH, 6 WRKY, 3 Asp, and 1 bZIP, were expressed in the leaf, while 3 bHLH and each one of MYB, WRKY, and Asp were regulated in the ovule. Above all, DELLA (LOC100280169) and Aspartic peroxidase (LOC100127531) genes are commonly up and down-regulated in the leaves and ovules of maize.

**Table 5:** Candidate genes conferring heat stress tolerance identified in previous studies in maize.

Stage	Tissue	Candidate genes	Reference
21 days old seedlings	Leaves	607 heat responsive genes, 39 heat tolerance genes	Frey et al. (2015)
6-day old seedlings	Leaves	Transcription factors (ERFs,NACs,ARF,MYB, andHD-ZIP)	Li et al. (2017)
Three-week-old seedlings	Leaves	Secondary metabolite biosynthetic pathway genes	Shi et al. (2017)
V3 and R1	Leaves, stalks, roots, tassels, ears, and silks	Pentatricopeptide repeat (PPR) proteins, miR168 and miR528	He et al. (2019)
Three-week-old seedlings (five-leaf stage)	Leaves	Heat shock proteins (Hsp40, Hsp70, Hsp90, Hsp100, and small Hsps) and Transcription factors (AP2-EREBP, MYB, bHLH, b-ZIP, and WRKY)	Qian et al. (2019)
V3	Leaves	Spliceosome metabolic pathways	Zhao et al. (2019)
V3	Leaves	Protein Kinases (35 CDPK, 9 MAPK, 53 serine/threonine-protein kinase), 6 aquaporin, 3 anion channnel protein, 41 MFS transporter, 168 TF, 94 HSPs, 1 prolinerich protein, 25 POX, 28 E3 ubiquitin-protein ligase, 24 ubiquitin-conjugating enzyme E2, 12 auxin-responsive protein IAA, and 3 abscisic acid receptor PYR/PYL family	Wu et al. (2020)
2-5-day-old seedlings	Etiolated coleoptiles	Protein renaturation, Biomembrane repair, Osmotic adjustment, and Redox balance	Li and Ye (2022)
R1	Flag leaf, pollen, ovule	Transcription Factors (AP2, MYB, WRKY, PsbP, bZIP, and NAM), heat shock proteins (HSP20, HSP70, and HSP101/ClpB), photosynthesis genes(PsaD&PsaN), antioxidation (APX and CAT) and polyamines (Spd and Spm).	Present study

Contrastingly, we observed tissue-specific differential expression of a few TFs, SBP, NAM, Exo70, and YABBY, which are mainly expressed at reproductive stages. These TFs were specific to leaf, pollen, or ovule. In our study, SBP was exclusively expressed in the leaf, which plays a critical role in many biological processes, notably flower development (Li et al., 2019). Also, we found that NAM and Exo70 TFs were expressed only in pollen. The NAM is associated with pollen and tapetum development in biotic and abiotic stress responses (Yang et al., 2018). The Exo70 plays a vital role in the exocyst complex function for pollen development, pollen grain germination, and pollen tube elongation (Synek et al., 2017). We also reported that the YABBY TF (a subfamily of ZF TF) was up-regulated in the ovule. It is basically involved in lateral organ development, dorsoventral polarity, and abiotic stress responses (Zhang et al., 2019).

### Shifts in energy metabolism and signal transduction plays crucial role in heat stress tolerance

Heat stress prompts the formation of reactive nitrogen species (RNS like NO) and reactive oxygen species (ROS), such as OH−, H_2_O_2_, and O_2_−, resulting in increased electrolyte leakage and lipid peroxidation. It led to enhance activities of antioxidant enzymes. Among them, the superoxide radical (O_2_−) is dismuted by superoxide dismutase (SOD) into H_2_O_2_ and further scavenged by catalase (CAT) and peroxidases (such as POD) through converting into H_2_O (Alam et al., 2017). Various studies reported that the cytochromes P450-related genes were expressed differentially under heat stress conditions in response to increased concentrations of ROS (Frey et al., 2015; Wang et al., 2016). In the present study, many oxidative metabolism-related genes like cytochromes P450 were up-regulated, confirming that ROS are generated during heat stress.

Heat stress affects plant photosynthesis negatively by inactivating photosystems, PSII, and PSI (Mathur et al., 2014). Photochemical reactions in thylakoid lamellae and carbon metabolism in chloroplast stroma have been noticed as the injury sites under heat stress (Wahid et al., 2007). DEGs related to PSII and PSI were up-regulated during heat stress in the present study. Several studies reported differential expression of genes encoding photosynthetic electron transfer- Cytb6/f, and PSII subunits, PsaD and PsaN, in response to heat stress (Wang et al., 2016; Wang et al., 2017). Similarly, dehydrins (DHNs) are a family of plant proteins induced in response to abiotic stresses or during later stages of embryogenesis (Close, 1997). The DHNs are highly hydrophilic and thermostable and act as chaperones that bind to calcium and help in water storage to impede cells from excessive dehydration (Hara et al., 2001; Yu et al., 2018). In the present investigation, DHNs were up-regulated in the ovule in response to heat stress. Tissue-specific expression and functional role of dehydrins per heat tolerance of C4 sugarcane (*Saccharum officinarum*) were confirmed by Galani et al. (2013). Several studies showed the role of dehydrins in abiotic stress tolerance in plants by stabilizing membranes, enzymes, and nucleotides in cells (Yu et al., 2018; Liu et al., 2019). The activation of genes involved in photosystem, antioxidants, and lipid peroxidation could be related to the maintenance of membrane integrity to confer heat tolerance.

### Role of hormones in signaling cascades of heat stress

Under heat stress, hormone homeostasis is altered, including hormone stability, biosynthesis, total contents, and compartmentalization (Davies, 2004). Although the involvement of hormones in plant heat resilience is complex, the signal pathway of hormones is not yet elucidated under heat stress. Many studies have delineated that optimizing certain hormones can enhance heat resilience in plants (Kotak et al., 2007; Wahid et al., 2007). Li et al. (2015) revealed that hormones such as ABA, auxin, jasmonic acid (JA), cytokinins (CKs), ethylene, gibberellin, and brassinosteroid are likely to be involved in heat stress tolerance. Several hormones, including ABA, brassinosteroids (BRs), and ethylene, possibly interacted through complex networks to regulate heat stress responses (Qu et al., 2013). In the present study, we found that DEGs related to P450 were up-regulated that involved in brassinosteroid biosynthesis. Contrastingly, DEG encoding remorin was down-regulated and negatively regulated the brassinosteroid mediated signaling pathway. Likewise, the ZIM motif family protein encoding was differentially expressed for pollen development under heat stress. JASMONATE ZIM-domain (JAZ) subfamily proteins have a role in biological processes such as development, stress-related, and hormone responses in *Arabidopsis*, rice, chickpea, and grape (Saha et al., 2016). Interestingly, both up-and down-regulated genes involved in hormone response pathways were identified, indicating that genes might help these pathways to keep the homeostasis under heat stress.

### Induction of HSPs under heat stress-a natural phenomenon

HSP genes such as HSP70, HSP101/ClpB, and HSP20 were induced during heat stress. HSP20 was up-regulated in all three tissues, whereas HSP101/ClpB and HSP70 were up-regulated in pollen and ovule, respectively. Extensive studies have demonstrated the protection of HSP70, HSP101, HSP20, and sHSPs family proteins from heat stress (Wang et al., 2016; Wang et al., 2017; Guo et al., 2019; Raju et al., 2020). Clp proteins belong to the large AAA+ (ATPases associated with diverse cellular activities) superfamily proteins. Furthermore, several studies illustrated that hydrogen peroxide (H_2_O_2_) generated during heat stress could enhance the ABA-dependent expression of HSP70 and sHSPs to tolerate heat stress (Liu et al., 2013; Li et al., 2014; Zhang et al., 2015). Therefore, the higher levels of expression are to acclimatize heat stress. It indicates that HSPs act as a molecular chaperone that prevents protein aggregation and denaturation resulting in maintaining protein structure. Further, heat stress induces membrane fluidity which activates lipid signaling and affects the Ca2+ channels and antioxidants. Therefore, increased lipid saturation is an important aspect of maintaining membrane fluidity. These cascades of reactions act as a primary signal for the activation of heat stress tolerance.

### Synthesis of polyamines

S-adenosylmethionine decarboxylase (SAMDC) is a key enzyme controlling the rate of polyamines formation that plays a pivotal role in plant growth, development, and adaptation to abiotic stresses (Mellidou et al., 2016). In the present investigation, the gene encoding for SAMDC showed up and down-regulation in all three tissues. Heat stress at 38°C and above suppressed the SAMDC activity, resulting in impaired polyamine biosynthesis and inhibition of pollen germination (Chen et al., 2015). It may be likely the cause of reduced pollen viability in heat-susceptible inbred LM 11 under high temperatures in the present study. The overexpression of the SAMDC gene resulted in elevated levels of Spd and/or Spm and enhanced the plant tolerance to abiotic stresses (Chen et al., 2015). Song et al. (2002) reported that suppression of SAMDC activity is a significant cause of inhibition of pollen germination and tube growth in tomatoes at high-temperature. Further, the essential role of Spd and Spm in pollen viability and seed development has been studied by Chen et al. (2015).

The present study ascertained that the metabolic overview pathway and secondary metabolites biosynthesis pathway, with the involvement of 264 and 146 genes, respectively, were the top two metabolic pathways in response to heat stress. Also, carbon metabolism, starch, sucrose metabolism, biosynthesis of amino acids, and proteins processing in the endoplasmic reticulum and plant hormone signal transduction are the most enriched pathways under heat stress. Shi et al. (2017) reported the expression of proteins under heat stress in sweet maize involved in a series of biological processes from translation to metabolic pathways and secondary metabolite synthesis. During heat stress, the seed response recorded in *Brassica napus* stated that heat treatment specifically affects the pathways, including ribosome, biosynthesis of amino acids, starch and sucrose metabolism, and protein processing in the endoplasmic reticulum and carbon metabolism (Guo et al., 2019). The upregulation of the genes involved in these pathways indicates that a set of genes are regulated to minimize protein modification during heat stress and impart tolerance in CML 25. The detected candidate genes could be exploited in maize heat-resilience breeding programs.

## Conclusion

The comparative transcriptomic studies in CML 25 and LM 11 maize inbreds under heat stress at the reproductive stage from three tissues (leaf, pollen, and ovule) gave insights into tissue-specific stress-related genes involved in biological pathways like metabolic processes, secondary metabolite synthesis, starch, and sucrose metabolism, carbon metabolism, protein synthesis, etc. Most DEGs were transcription factors, heat shock proteins, antioxidants, hormone biosynthesis, and polyamine biosynthesis-related genes. Seven DEGs were common in leaf, pollen, and ovule; and involved in the polyamines biosynthesis pathway that could be further explored in understanding heat tolerance mechanism. The up-regulated genes identified in heat-tolerant inbred CML 25 would be the potential candidate genes that could be utilized for the development of heat-resilient maize using marker-assisted backcross breeding.

## Data availability statement

The original contributions presented in the study are publicly available. This data can be found here: NCBI, PRJNA656908.

## Author contributions

Conceptualization: YV and GJ; Project administration and resources: YV; Methodology: AJ, YV, and IY; Bioinformatics analysis: AJ and IY; Methodology: IY and AJ; Validation: NK and AS; Original draft: AJ, UP, and YV; Finalized the manuscript: AJ, YV, IY, and GJ. All authors contributed to the article and approved the submitted version. 

## Supplementary material

Supplementary Figure 1 Principal component analysis (PCA plot) of all RNA-seq maize samples at reproductive stage under heat stress.

Supplementary Figure 2 Heatmap of differentially expressed genes (DEGs) in response to heat stress. DEGs have been identified with significant (P *<* 0.05) and |log_2_ (fold change)| ≥2 in leaf, pollen and ovule of CML 25 (HT) and LM 11 (HS) inbred. Colors represent log_2_ up-regulation (yellow) and down-regulation (blue) of DEGs.
