# Supplementary figures and images for: Transcriptional dynamics of maize leaves, pollens and ovules to gain insights into heat stress-related responses
Source: Front Plant Sci. 2023 Feb 15;14:1117136. doi: 10.3389/fpls.2023.1117136 (PMC9975602; doi:10.3389/fpls.2023.1117136)

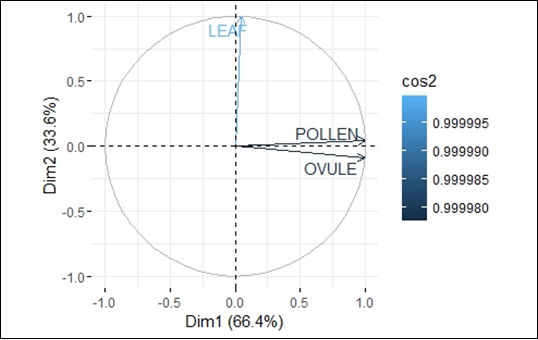

Supplement: Supplementary Figure 1 — Principal component analysis (PCA plot) of all RNA-seq maize samples at reproductive stage under heat stress. [file Image_1.tif]

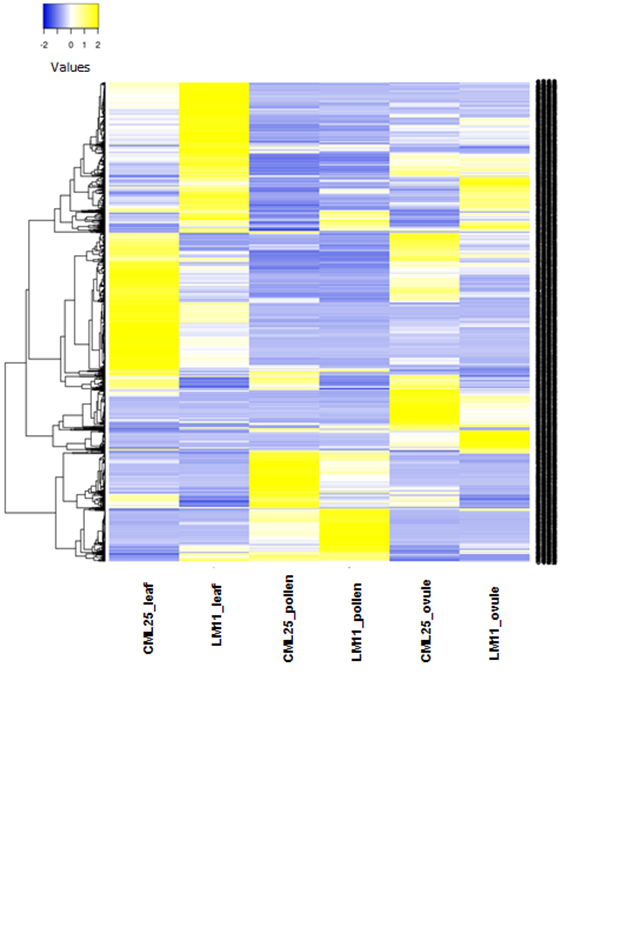

Supplement: Supplementary Figure 2 — Heatmap of differentially expressed genes (DEGs) in response to heat stress. DEGs have been identified with significant (P < 0.05) and |log2 (fold change)| ≥2 in leaf, pollen and ovule of CML 25 (HT) and LM 11 (HS) inbred. Colors represent log2 up-regulation (yellow) and down-regulation (blue) of DEGs. [file Image_2.png]
