# Supplementary material for: Transcriptional dynamics of maize leaves, pollens and ovules to gain insights into heat stress-related responses
Source: Front Plant Sci. 2023 Feb 15;14:1117136. doi: 10.3389/fpls.2023.1117136 (PMC9975602; doi:10.3389/fpls.2023.1117136)
Supplement: Supplementary file 3 [file Table_1.docx]

**Supplementary Table S1** GO enrichment of differentially expressed genes (DEGs) in leaf under heat stress conditions.

| **GO term** | **GO category** | **Number of DEGs** | **Percentage of DEGs** | **Descriptions** |
| --- | --- | --- | --- | --- |
| GO:0005623 | CC | 568 | 58.8 | cell |
| GO:0044464 | CC | 564 | 58.4 | cell part |
| GO:0044422 | CC | 189 | 19.6 | organelle part |
| GO:0043226 | CC | 394 | 40.8 | organelle |
| GO:0032991 | CC | 87 | 9 | macromolecular complex |
| GO:0044425 | CC | 335 | 34.7 | membrane part |
| GO:0016020 | CC | 401 | 41.5 | membrane |
| GO:0031974 | CC | 22 | 2.3 | membrane-enclosed lumen |
| GO:0099080 | CC | 1 | 0.1 | supramolecular complex |
| GO:0005576 | CC | 27 | 2.8 | extracellular region |
| GO:0044421 | CC | 7 | 0.7 | extracellular region part |
| GO:0055044 | CC | 12 | 1.2 | symplast |
| GO:0030054 | CC | 12 | 1.2 | cell junction |
| GO:0009295 | CC | 3 | 0.3 | nucleoid |
| GO:0003824 | MF | 535 | 55.4 | catalytic activity |
| GO:0005488 | MF | 490 | 50.7 | binding |
| GO:0140110 | MF | 51 | 5.3 | transcription regulator activity |
| GO:0016209 | MF | 20 | 2.1 | antioxidant activity |
| GO:0004871 | MF | 12 | 1.2 | signal transducer activity |
| GO:0060089 | MF | 8 | 0.8 | molecular transducer activity |
| GO:0005215 | MF | 79 | 8.2 | transporter activity |
| GO:0098772 | MF | 13 | 1.3 | molecular function regulator |
| GO:0005198 | MF | 17 | 1.8 | structural molecule activity |
| GO:0008152 | BP | 626 | 64.8 | metabolic process |
| GO:0009987 | BP | 561 | 58.1 | cellular process |
| GO:0051179 | BP | 117 | 12.1 | localization |
| GO:0032502 | BP | 38 | 3.9 | developmental process |
| GO:0032501 | BP | 31 | 3.2 | multicellular organismal process |
| GO:0071840 | BP | 80 | 8.3 | cellular component organization or biogenesis |
| GO:0050789 | BP | 176 | 18.2 | regulation of biological process |
| GO:0065007 | BP | 197 | 20.4 | biological regulation |
| GO:0002376 | BP | 12 | 1.2 | immune system process |
| GO:0050896 | BP | 209 | 21.6 | response to stimulus |
| GO:0048519 | BP | 27 | 2.8 | negative regulation of biological process |
| GO:0098754 | BP | 24 | 2.5 | detoxification |
| GO:0051704 | BP | 28 | 2.9 | multi-organism process |
| GO:0048518 | BP | 21 | 2.2 | positive regulation of biological process |
| GO:0023052 | BP | 57 | 5.9 | signaling |
| GO:0022414 | BP | 20 | 2.1 | reproductive process |
| GO:0000003 | BP | 20 | 2.1 | reproduction |
| GO:0001906 | BP | 2 | 0.2 | cell killing |
| GO:0048511 | BP | 7 | 0.7 | rhythmic process |
| GO:0040007 | BP | 6 | 0.6 | growth |
| GO:0015976 | BP | 3 | 0.3 | carbon utilization |

**Supplementary Table S2** GO enrichment of differentially expressed genes (DEGs) in pollen under heat stress conditions.

| **GO term** | **GO category** | **Number of DEGs** | **Percentage of DEGs** | **Descriptions** |
| --- | --- | --- | --- | --- |
| GO:0044464 | CC | 188 | 54.8 | cell part |
| GO:0005623 | CC | 189 | 55.1 | cell |
| AGO:0044422 | CC | 45 | 13.1 | organelle part |
| GO:0043226 | CC | 101 | 29.4 | organelle |
| GO:0032991 | CC | 23 | 6.7 | macromolecular complex |
| GO:0031974 | CC | 12 | 3.5 | membrane-enclosed lumen |
| GO:0044425 | CC | 105 | 30.6 | membrane part |
| GO:0016020 | CC | 138 | 40.2 | membrane |
| GO:0099080 | CC | 3 | 0.9 | supramolecular complex |
| GO:0055044 | CC | 3 | 0.9 | symplast |
| GO:0030054 | CC | 3 | 0.9 | cell junction |
| GO:0005576 | CC | 18 | 5.2 | extracellular region |
| GO:0044421 | CC | 5 | 1.5 | extracellular region part |
| GO:0003824 | MF | 199 | 58 | catalytic activity |
| GO:0005198 | MF | 7 | 2 | structural molecule activity |
| GO:0005488 | MF | 147 | 42.9 | binding |
| GO:0005215 | MF | 18 | 5.2 | transporter activity |
| GO:0004871 | MF | 15 | 4.4 | signal transducer activity |
| GO:0060089 | MF | 14 | 4.1 | molecular transducer activity |
| GO:0140110 | MF | 9 | 2.6 | transcription regulator activity |
| GO:0016209 | MF | 2 | 0.6 | antioxidant activity |
| GO:0098772 | MF | 19 | 5.5 | molecular function regulator |
| GO:0045735 | MF | 8 | 2.3 | nutrient reservoir activity |
| GO:0032502 | BP | 12 | 3.5 | developmental process |
| GO:0032501 | BP | 11 | 3.2 | multicellular organismal process |
| GO:0022414 | BP | 13 | 3.8 | reproductive process |
| GO:0000003 | BP | 13 | 3.8 | reproduction |
| GO:0009987 | BP | 185 | 53.9 | cellular process |
| GO:0071840 | BP | 38 | 11.1 | cellular component organization or biogenesis |
| GO:0008152 | BP | 193 | 56.3 | metabolic process |
| GO:0051179 | BP | 31 | 9 | localization |
| GO:0050789 | BP | 42 | 12.2 | regulation of biological process |
| GO:0065007 | BP | 69 | 20.1 | biological regulation |
| GO:0050896 | BP | 60 | 17.5 | response to stimulus |
| GO:0098754 | BP | 2 | 0.6 | detoxification |
| GO:0048519 | BP | 5 | 1.5 | negative regulation of biological process |
| GO:0023052 | BP | 25 | 7.3 | signaling |
| GO:0051704 | BP | 6 | 1.7 | multi-organism process |
| GO:0048518 | BP | 1 | 0.3 | positive regulation of biological process |

**Supplementary Table S3** GO enrichment of differentially expressed genes (DEGs) in ovule under heat stress conditions.

| **GO term** | **GO category** | **Number of DEGs** | **Percentage of DEGs** | **Descriptions** |
| --- | --- | --- | --- | --- |
| GO:0044464 | CC | 248 | 55.7 | cell part |
| GO:0005623 | CC | 249 | 56 | cell |
| GO:0043226 | CC | 174 | 39.1 | organelle |
| GO:0031974 | CC | 17 | 3.8 | membrane-enclosed lumen |
| GO:0044422 | CC | 80 | 18 | organelle part |
| GO:0032991 | CC | 62 | 13.9 | macromolecular complex |
| GO:0016020 | CC | 179 | 40.2 | membrane |
| GO:0044425 | CC | 154 | 34.6 | membrane part |
| GO:0099080 | CC | 2 | 0.4 | supramolecular complex |
| GO:0005576 | CC | 30 | 6.7 | extracellular region |
| GO:0044421 | CC | 6 | 1.3 | extracellular region part |
| GO:0055044 | CC | 11 | 2.5 | symplast |
| GO:0030054 | CC | 11 | 2.5 | cell junction |
| GO:0003824 | MF | 217 | 48.8 | catalytic activity |
| GO:0005488 | MF | 213 | 47.9 | binding |
| GO:0016209 | MF | 11 | 2.5 | antioxidant activity |
| GO:0005215 | MF | 40 | 9 | transporter activity |
| GO:0140110 | MF | 12 | 2.7 | transcription regulator activity |
| GO:0004871 | MF | 5 | 1.1 | signal transducer activity |
| GO:0060089 | MF | 2 | 0.4 | molecular transducer activity |
| GO:0098772 | MF | 15 | 3.4 | molecular function regulator |
| GO:0045735 | MF | 7 | 1.6 | nutrient reservoir activity |
| GO:0031386 | MF | 1 | 0.2 | protein tag |
| GO:0005198 | MF | 5 | 1.1 | structural molecule activity |
| GO:0140104 | MF | 1 | 0.2 | molecular carrier activity |
| GO:0050896 | BP | 96 | 21.6 | response to stimulus |
| GO:0008152 | BP | 246 | 55.3 | metabolic process |
| GO:0009987 | BP | 241 | 54.2 | cellular process |
| GO:0051179 | BP | 73 | 16.4 | localization |
| GO:0065007 | BP | 76 | 17.1 | biological regulation |
| GO:0032502 | BP | 29 | 6.5 | developmental process |
| GO:0032501 | BP | 26 | 5.8 | multicellular organismal process |
| GO:0022414 | BP | 23 | 5.2 | reproductive process |
| GO:0000003 | BP | 23 | 5.2 | reproduction |
| GO:0051704 | BP | 18 | 4 | multi-organism process |
| GO:0050789 | BP | 60 | 13.5 | regulation of biological process |
| GO:0048519 | BP | 17 | 3.8 | negative regulation of biological process |
| GO:0002376 | BP | 1 | 0.2 | immune system process |
| GO:0071840 | BP | 55 | 12.4 | cellular component organization or biogenesis |
| GO:0098754 | BP | 12 | 2.7 | detoxification |
| GO:0023052 | BP | 15 | 3.4 | signaling |
| GO:0048518 | BP | 4 | 0.9 | positive regulation of biological process |
| GO:0040007 | BP | 4 | 0.9 | growth |
| GO:0048511 | BP | 1 | 0.2 | rhythmic process |
| GO:0008283 | BP | 1 | 0.2 | cell proliferation |
| GO:0001906 | BP | 2 | 0.4 | cell killing |

**Supplementary Table S4** Details of transcription factors (TFs) exclusively expressed in leaf under heat stress conditions.

| **Transcript Id** | **Log_2_ Fold Change** | **Transcription Factors (TFs)** | **Description** |
| --- | --- | --- | --- |
| comp79188_c1_seq8 | 2.99 | zf-C2H2_6 | predicted protein |
| comp76955_c0_seq53 | 4.74 | WRKY | TPA: putative WRKY DNA-binding domain superfamily protein |
| comp76955_c0_seq51 | 4.00 | WRKY | TPA: putative WRKY DNA-binding domain superfamily protein |
| comp81491_c0_seq22 | 3.66 | WRKY | probable WRKY transcription factor 65 |
| comp80874_c0_seq18 | 3.55 | WRKY | probable WRKY transcription factor 65 |
| comp76955_c0_seq22 | -3.50 | WRKY | WRKY74 - superfamily of TFs having WRKY and zinc finger domains |
| comp76955_c0_seq43 | -3.96 | WRKY | TPA: putative WRKY DNA-binding domain superfamily protein |
| comp86201_c0_seq2 | -3.53 | TCP, Cellulose_synt | cellulose synthase-like CslF6 |
| comp14208_c0_seq1 | 3.67 | PsbP | psbP domain-containing protein 3, chloroplastic |
| comp58181_c0_seq1 | 3.60 | PsbP | psbP domain-containing protein 3, chloroplastic |
| comp76476_c0_seq2 | 3.57 | PsbP | PsbP domain-containing protein 1, chloroplastic |
| comp60981_c0_seq1 | 3.05 | PsbP | psbP domain-containing protein 6, chloroplastic |
| comp81869_c0_seq5 | 4.61 | PAP2 | lipid phosphate phosphatase epsilon 2, chloroplastic-like |
| comp80309_c0_seq8 | 3.09 | Myb_DNA-binding,Myb_CC_LHEQLE | protein PHR1-LIKE 1-like |
| comp84639_c0_seq16 | 4.92 | Myb_DNA-binding | transcription factor MYB59-like |
| comp21387_c0_seq2 | -3.16 | Myb_DNA-binding | putative MYB DNA-binding domain superfamily protein |
| comp85415_c0_seq8 | -3.46 | Myb_DNA-binding | protein ODORANT1-like |
| comp85878_c1_seq7 | -3.51 | Myb_DNA-binding | LHY protein isoform X1 |
| comp81339_c2_seq11 | -4.24 | Myb_DNA-binding | myb-related protein P-like |
| comp80297_c1_seq4 | -3.04 | Myb_DNA-bind_6 | myb-related protein P-like |
| comp75648_c0_seq3 | -3.85 | Myb_DNA-bind_4 | myb-related protein P-like |
| comp71320_c0_seq4 | 3.55 | Myb_CC_LHEQLE | putative MYB DNA-binding domain superfamily protein |
| comp83381_c0_seq7 | 4.07 | mTERF | predicted protein |
| comp83381_c0_seq6 | 3.97 | mTERF | predicted protein |
| comp80495_c0_seq2 | 3.24 | mTERF | predicted protein |
| comp84014_c0_seq3 | 2.96 | DELLA, GRAS | reduced height-2 |
| comp83970_c0_seq2 | -5.21 | DELLA, GRAS | reduced height-2 |
| comp87149_c0_seq9 | 4.82 | CPP1-like | protein phosphatase 2C |
| comp87267_c0_seq2 | -3.78 | Cpn60_TCP1 | mitochondrial chaperonin-60 |
| comp79361_c0_seq2 | -2.97 | bZIP_1 | putative bZIP transcription factor superfamily protein |
| comp65725_c0_seq1 | -3.06 | bZIP_1 | transcription factor HY5 |
| comp72312_c0_seq2 | -3.36 | bZIP_1 | putative bZIP transcription factor superfamily protein |
| comp85029_c0_seq6 | -3.48 | bZIP_1 | transcription factor HY5 |
| comp72776_c0_seq6 | -3.62 | bZIP_1 | ocs element-binding factor 1-like |
| comp82575_c1_seq4 | -3.78 | bZIP_1 | putative bZIP transcription factor superfamily protein |
| comp82575_c1_seq12 | -4.05 | bZIP_1 | putative bZIP transcription factor superfamily protein isoform X1 |
| comp79361_c0_seq1 | -4.28 | bZIP_1 | putative bZIP transcription factor superfamily protein |
| comp79361_c0_seq4 | -4.44 | bZIP_1 | transcription factor HY5-like |
| comp85804_c0_seq5 | -3.90 | bHLH-MYC_N, HLH | transcription factor MYC4-like |
| comp69541_c0_seq2 | -3.67 | BES1_N | protein BZR1 homolog 1-like |
| comp85282_c0_seq6 | -3.47 | B3, AUX_IAA | auxin response factor 4 |
| comp70437_c0_seq2 | -3.26 | B3 | exocyst complex component EXO70A1-like |
| comp84387_c0_seq3 | 3.41 | Asp, SapB_2, SapB_1 | aspartic proteinase oryzasin-1-like |
| comp69314_c0_seq2 | -3.46 | Asp, SapB_2 | aspartic proteinase |
| comp70287_c0_seq2 | -3.53 | Asp, SapB_2 | aspartic proteinase |
| comp70059_c0_seq2 | -3.15 | ArfGap | probable ADP-ribosylation factor GTPase-activating protein AGD8 |
| comp83741_c1_seq17 | 5.22 | AP2 | ethylene-responsive transcription factor 3 |
| comp71391_c0_seq2 | 3.76 | AP2 | ethylene-responsive element binding protein 2 |
| comp71391_c0_seq1 | 3.43 | AP2 | ethylene-responsive element binding protein 2 |
| comp79428_c3_seq5 | 3.39 | AP2 | ethylene-responsive transcription factor 4 |
| comp45633_c0_seq1 | -3.33 | AP2 | ethylene-responsive transcription factor ERF020-like |
| comp84050_c0_seq8 | -4.11 | AP2 | C-repeat binding factor 3 |
| comp81165_c1_seq3 | -4.21 | AP2 | AP2-EREBP transcription factor |
| comp83960_c0_seq8 | 3.74 | ANF_receptor, SBP_bac_3, Lig_chan | glutamate receptor 3.1 |
| comp83960_c0_seq6 | -4.61 | ANF_receptor, SBP_bac_3, Lig_chan | glutamate receptor 3.1 |

**Supplementary Table S5** Details of transcription factors (TFs) exclusively expressed in pollen under heat stress conditions.

| **Transcript Id** | **Log_2_ Fold Change** | **Transcription Factors (TFs)** | **Description** |
| --- | --- | --- | --- |
| comp79006_c0_seq2 | -5.34 | ArfGap-C2 | probable ADP-ribosylation factor GTPase-activating protein AGD11 isoform X1 |
| comp84652_c1_seq8 | -4.20 | NAM | NAC domain-containing protein 68 |
| comp80690_c0_seq4 | 4.06 | WRKY | probable WRKY transcription factor 47 |
| comp82481_c1_seq3 | -4.07 | zf-C2H2_6 | predicted protein |
| comp79716_c1_seq1 | -3.69 | ArfGap-C2 | probable ADP-ribosylation factor GTPase-activating protein AGD11 isoform X1 |
| comp76234_c1_seq23 | -3.61 | Myb_DNA-bind_6 | putative MYB DNA-binding domain superfamily protein |
| comp80337_c0_seq3 | -3.33 | zf-C3HC4_3 | RING finger protein 5 |
| comp84564_c0_seq21 | -3.52 | zf-C3HC4_3 | RING finger protein 5 |
| comp84050_c0_seq2 | 3.31 | Exo70,AP2 | exocyst complex component EXO70B1-like |

**Supplementary Table S6** Details of transcription factors (TFs) exclusively expressed in ovule under heat stress conditions.

| **Transcript Id** | **Log_2_ Fold Change** | **Transcription Factors (TFs)** | **Description** |
| --- | --- | --- | --- |
| comp70287_c0_seq2 | -5.44 | Asp, SapB_2 | aspartic proteinase |
| comp82434_c0_seq3 | 4.26 | AP2 | ethylene-responsive transcription factor 3 |
| comp84014_c0_seq3 | 4.46 | DELLA, GRAS | reduced height-2 |
| comp76315_c0_seq13 | 3.92 | YABBY | TPA: putative YABBY domain transcription factor family protein |
| comp77908_c2_seq1 | -3.83 | bZIP_1 | transcription factor RF2b-like |
| comp85721_c0_seq3 | -3.85 | Cpn60_TCP1 | T-complex protein 1 subunit epsilon |
| comp85835_c0_seq2 | 3.92 | Cpn60_TCP1 | T-complex protein 1 subunit epsilon |
| comp85683_c1_seq4 | -3.85 | WRKY | WRKY transcription factor |
| comp77778_c0_seq1 | -3.80 | Cpn60_TCP1 | chaperonin CPN60-like 2, mitochondrial |
| comp80921_c0_seq8 | 3.37 | Myb_DNA-binding | DNA binding protein |
| comp67382_c0_seq1 | 3.61 | Myb_DNA-bind_6, Myb_Cef | cell division cycle 5-like protein |
| comp79882_c0_seq7 | 3.38 | Arf | ADP-ribosylation factor 1 |
| comp82606_c0_seq5 | -3.40 | bZIP_1 | transcription factor RF2b-like |
| comp80888_c0_seq4 | -3.32 | bZIP_1 | transcription factor RF2b-like |

**Supplementary Table S7** Details of up-regulated differentially expressed transcripts related to metabolic processes in leaf under heat stress conditions.

| **Transcript Id** | **Log_2_ Fold Change** | **Transcript description** |
| --- | --- | --- |
| comp74270_c0_seq1 | 7.13 | ascorbate peroxidase |
| comp71063_c0_seq5 | 6.78 | stress responsive protein |
| comp85856_c3_seq9 | 6.87 | bidirectional sugar transporter SWEET13-like |
| comp65718_c0_seq1 | 6.34 | alpha-dioxygenase 1-like |
| comp65718_c1_seq1 | 5.62 | alpha-dioxygenase 1-like |
| comp77833_c1_seq33 | 4.61 | peroxidase 5-like |
| comp79476_c4_seq4 | 4.26 | peroxidase 5-like |
| comp85849_c2_seq8 | 3.55 | putative L-ascorbate peroxidase 6 isoform X1 |
| comp83226_c1_seq1 | 7.16 | granule-bound starch synthase II |
| comp85923_c1_seq18 | 6.53 | ABA 8'-hydroxylase |
| comp75508_c0_seq7 | 6.38 | tab2 protein |
| comp84970_c0_seq11 | 6.20 | photosynthetic NDH subunit of subcomplex B 2, chloroplastic |
| comp77362_c1_seq2 | 5.74 | catalase |
| comp72479_c0_seq1 | 5.53 | predicted protein |
| comp82556_c0_seq1 | 5.69 | predicted protein |
| comp76644_c0_seq1 | 5.32 | photosystem I reaction center subunit II |
| comp65468_c0_seq2 | 5.35 | photosystem I reaction center subunit N, chloroplastic-like |
| comp73293_c0_seq2 | 5.06 | Thiazole biosynthetic enzyme Thi4 family |
| comp83408_c0_seq1 | 5.04 | zeaxanthin 7,8(7',8')-cleavage dioxygenase, chromoplast |
| comp64508_c0_seq1 | 5.15 | TPA: putative cinnamyl-alcohol dehydrogenase family protein |
| comp84863_c0_seq1 | 9.66 | cytochrome b6-f complex iron-sulfur subunit |

**Supplementary Table S8** Details of down-regulated differentially expressed transcripts related to metabolic processes in leaf under heat stress conditions.

| **Transcript Id** | **Log_2_ Fold Change** | **Transcript description** |
| --- | --- | --- |
| comp62622_c0_seq1 | -6.07 | leucoanthocyanidin dioxygenase-like |
| comp87483_c0_seq1 | -6.01 | naringenin,2-oxoglutarate 3-dioxygenase-like |
| comp87489_c0_seq1 | -5.97 | naringenin,2-oxoglutarate 3-dioxygenase-like |
| comp61430_c0_seq2 | -5.89 | leucoanthocyanidin dioxygenase-like |
| comp83685_c4_seq5 | -5.68 | membrane protein |
| comp83685_c0_seq6 | -5.57 | membrane protein |
| comp57152_c0_seq1 | -5.49 | dirigent-like protein pDIR3 isoform X1 |
| comp85462_c1_seq5 | -5.32 | catalytic/ hydrolase |
| comp85370_c0_seq1 | -5.25 | probable threonine--tRNA ligase, cytoplasmic |
| comp81888_c0_seq3 | -5.11 | 70 kDa peptidyl-prolyl isomerase |
| comp87665_c0_seq1 | -4.96 | very-long-chain enoyl-CoA reductase-like |
| comp83576_c0_seq29 | -4.94 | high-light-induced protein, chloroplastic-like |
| comp37119_c0_seq1 | -4.84 | very-long-chain enoyl-CoA reductase-like |
| comp86722_c0_seq3 | -4.72 | cytochrome P450 94C1-like |
| comp81721_c0_seq33 | -4.68 | carotenoid hydroxylase |
| comp85015_c0_seq7 | -4.25 | chlorophyll a-b binding protein of LHCII type 1-like |
| comp84544_c0_seq11 | -4.11 | cytochrome P450 94C1-like |
| comp65496_c0_seq2 | -4.10 | predicted protein |
| comp58210_c0_seq1 | -4.08 | lysM domain containing protein |
| comp57045_c0_seq1 | -4.03 | lysM domain containing protein |
| comp86722_c0_seq5 | -3.97 | predicted protein |
| comp84544_c0_seq1 | -3.93 | cytochrome P450 94C1-like |
| comp71565_c0_seq2 | -3.81 | delta(1)-pyrroline-2-carboxylate reductase |
| comp68536_c0_seq1 | -3.78 | putative alcohol dehydrogenase superfamily protein |
| comp77659_c0_seq1 | -3.48 | predicted protein |
| comp86585_c1_seq3 | -3.44 | probable pectinesterase/pectinesterase inhibitor 51 |
| comp68084_c2_seq1 | -3.38 | putative alcohol dehydrogenase superfamily protein |
| comp76443_c0_seq23 | -3.31 | psbP domain-containing protein 7, chloroplastic |
| comp75253_c0_seq2 | -3.16 | peroxidase 1-like |
| comp77346_c1_seq10 | -9.95 | dehydrin COR410 |
| comp83476_c0_seq4 | -7.02 | - |
| comp76644_c0_seq9 | -6.30 | photosystem I reaction center subunit II |
| comp72119_c1_seq2 | -7.37 | glycerate dehydrogenase |
| comp76807_c0_seq5 | -6.52 | NADP-dependent malic enzyme |

**Supplementary Table S9** Details of up-regulated differentially expressed transcripts related to metabolic processes in pollen under heat stress conditions.

| **Transcript Id** | **Log_2_ Fold Change** | **Transcript description** |
| --- | --- | --- |
| comp86232_c0_seq4 | 3.76 | heme oxygenase 1 |
| comp77808_c0_seq1 | 3.42 | transmembrane protein, putative |
| comp77040_c2_seq28 | 3.31 | cytochrome P450 90D2-like |
| comp78035_c0_seq5 | 3.51 | predicted protein |
| comp71657_c0_seq15 | 3.37 | autophagy-related protein 8 precursor |
| comp47513_c0_seq2 | 3.93 | NAD(P)-linked oxidoreductase superfamily protein isoform 1 |
| comp79192_c1_seq2 | 3.76 | cytochrome c oxidase subunit 6a, mitochondrial |
| comp79192_c0_seq1 | 3.31 | cytochrome c oxidase subunit 6a, mitochondrial |
| comp87307_c0_seq7 | 4.04 | pollen-specific protein NTP303 precursor |
| comp69718_c0_seq1 | 3.87 | uncharacterized protein LOC100303805 |
| comp86474_c1_seq3 | 3.83 | uncharacterized protein LOC100303805 |
| comp66116_c0_seq1 | 3.61 | probable NADH dehydrogenase [ubiquinone] 1 alpha subcomplex subunit 5, mitochondrial |
| comp80939_c0_seq2 | 8.59 | beta-amylase |
| comp79611_c0_seq6 | 3.26 | catalytic/ hydrolase/ phosphoglycolate phosphatase |
| comp79506_c1_seq1 | 3.52 | ATP phosphoribosyltransferase, chloroplastic-like |
| comp78590_c0_seq6 | 3.21 | copper-transporting ATPase RAN1-like |
| comp67304_c0_seq1 | 3.61 | glycerol-3-phosphate dehydrogenase [NAD(+)] |
| comp62906_c0_seq1 | 3.10 | isoflavone reductase-like protein |
| comp75013_c0_seq4 | 4.19 | ascorbate peroxidase |
| comp86409_c0_seq3 | 5.75 | TPA: kinase superfamily protein |
| comp81122_c0_seq4 | 3.89 | squalene monooxygenase |
| comp86409_c0_seq11 | 4.18 | U-box domain-containing protein 52 |
| comp83658_c0_seq1 | 3.48 | protein WEAK CHLOROPLAST MOVEMENT UNDER BLUE LIGHT 1-like |

**Supplementary Table S10** Details of down-regulated differentially expressed transcripts related to metabolic process in pollen under heat stress conditions.

| **Transcript Id** | **Log_2_ Fold Change** | **Transcript description** |
| --- | --- | --- |
| comp77945_c0_seq3 | -9.74 | zinc finger and C2 domain protein-like |
| comp79197_c3_seq1 | -7.77 | glucan endo-1,3-beta-glucosidase 8-like |
| comp81575_c0_seq3 | -6.01 | PI-PLC X domain-containing protein At5g67130-like |
| comp79266_c1_seq3 | -5.97 | putative LIM-type zinc finger domain family protein |
| comp76680_c0_seq2 | -5.63 | bifunctional monodehydroascorbate reductase and carbonic anhydrase nectarin-3-like |
| comp82567_c0_seq3 | -5.70 | fumarylacetoacetase |
| comp79006_c0_seq2 | -5.34 | probable ADP-ribosylation factor GTPase-activating protein AGD11 isoform X1 |
| comp85337_c0_seq4 | -5.26 | alkaline/neutral invertase CINV2-like |
| comp86311_c1_seq8 | -5.12 | pyrophosphate-energized vacuolar membrane proton pump |
| comp79708_c0_seq5 | -4.36 | predicted protein |
| comp85996_c0_seq4 | -4.34 | vacuolar protein sorting-associated protein 20 homolog 2-like |
| comp82048_c1_seq3 | -3.96 | RALF precursor |
| comp79240_c0_seq4 | -4.05 | acyl-[acyl-carrier-protein] desaturase |
| comp85368_c0_seq3 | -3.90 | 65-kDa microtubule-associated protein 3-like |
| comp70405_c0_seq2 | -3.66 | ZIM motif family protein |
| comp87209_c1_seq1 | -3.63 | 22 kDa zein protein |
| comp87085_c4_seq2 | -3.67 | zein-alpha PMS1 Precursor |
| comp80939_c4_seq2 | -9.62 | beta-amylase 1, chloroplastic-like |
| comp80939_c4_seq6 | -6.67 | beta-amylase |

**Supplementary Table S11** Details of up-regulated differentially expressed transcripts related to metabolic process in ovule under heat stress conditions.

| **Transcript Id** | **Log_2_ Fold Change** | **Transcript description** |
| --- | --- | --- |
| comp61784_c0_seq2 | 7.90 | cysteine proteinase inhibitor 8-like |
| comp87411_c0_seq1 | 7.28 | Bowman-Birk type trypsin inhibitor |
| comp41080_c0_seq2 | 5.50 | protein ELC-like |
| comp85699_c1_seq5 | 5.43 | UDP-glucose 6-dehydrogenase, putative, expressed |
| comp68481_c0_seq1 | 5.20 | E3 ubiquitin-protein ligase XB3 |
| comp77520_c0_seq2 | 5.19 | ADP,ATP carrier protein 1, mitochondrial-like |
| comp86752_c1_seq2 | 4.96 | mitochondrial uncoupling protein 5-like |
| comp69330_c0_seq2 | 4.88 | nonspecific lipid-transfer protein 2 precursor |
| comp77030_c0_seq1 | 4.86 | type 1 non-specific lipid transfer protein precursor |
| comp81007_c0_seq9 | 4.73 | lipoxygenase 1 |
| comp77564_c1_seq1 | 4.72 | type 1 non-specific lipid transfer protein precursor |
| comp78918_c0_seq6 | 4.58 | V-type proton ATPase subunit D-like |
| comp18660_c0_seq1 | 4.43 | aldose reductase |
| comp67757_c0_seq2 | 4.42 | oleosin 18 kDa |
| comp85699_c0_seq4 | 4.39 | UDP-glucose 6-dehydrogenase, putative, expressed |
| comp44452_c0_seq1 | 4.35 | RAB17 protein |
| comp86784_c0_seq28 | 4.26 | GDSL esterase/lipase At5g45910-like |
| comp77833_c1_seq33 | 4.25 | peroxidase 5-like |
| comp81007_c0_seq5 | 4.18 | lipoxygenase 1 |
| comp36561_c0_seq1 | 4.14 | aldose reductase |
| comp81335_c0_seq1 | 4.09 | TPA: membrane H(+)-ATPase1 |
| comp70272_c0_seq1 | 3.99 | predicted protein |
| comp74342_c1_seq2 | 3.97 | cytochrome P450 81D11-like |
| comp85835_c0_seq2 | 3.92 | T-complex protein 1 subunit epsilon |
| comp77833_c1_seq14 | 3.88 | peroxidase 16-like |
| comp83570_c0_seq3 | 3.86 | diphosphomevalonate decarboxylase |
| comp72013_c0_seq1 | 3.84 | putative phosphoenolpyruvate carboxylase kinase family protein |
| comp72013_c0_seq1 | 3.84 | putative phosphoenolpyruvate carboxylase kinase family protein |
| comp67908_c0_seq1 | 3.82 | predicted protein |
| comp78859_c0_seq1 | 3.74 | multidomain cystatin |
| comp61084_c0_seq2 | 3.74 | uncharacterized membrane protein At3g27390 |
| comp72322_c0_seq3 | 3.72 | 11-beta-hydroxysteroid dehydrogenase-like 5 |
| comp75333_c0_seq2 | 3.69 | momilactone A synthase-like |
| comp13950_c0_seq1 | 3.60 | chitinase |
| comp13914_c0_seq1 | 3.60 | non-specific lipid-transfer protein A-like |
| comp87413_c0_seq1 | 3.57 | non-specific lipid-transfer protein A-like |
| comp82317_c0_seq2 | 3.53 | pyrophosphate--fructose 6-phosphate 1-phosphotransferase subunit alpha-like |
| comp82325_c0_seq10 | 3.52 | Serine carboxypeptidase-like 18 |
| comp79476_c4_seq7 | 3.49 | peroxidase 16-like |
| comp70274_c0_seq2 | 3.48 | deoxymugineic acid synthase1 |
| comp80184_c0_seq2 | 3.47 | predicted protein |
| comp55686_c0_seq1 | 3.44 | non-symbiotic hemoglobin |
| comp75016_c0_seq4 | 3.43 | GDSL esterase/lipase At5g45910-like |
| comp76805_c1_seq1 | 3.43 | calmodulin-7 |
| comp42139_c0_seq1 | 3.40 | 3-ketoacyl-CoA synthase 10-like |
| comp76136_c0_seq1 | 3.39 | thioredoxin H-type |
| comp73306_c0_seq3 | 3.38 | (+)-neomenthol dehydrogenase |
| comp78769_c0_seq1 | 3.21 | transcription elongation factor 1 homolog |
| comp13987_c0_seq1 | 3.08 | putative late embryogenesis abundant protein |
| comp82242_c0_seq4 | 3.11 | oleosin 18 kDa |

**Supplementary Table S12** Details of down-regulated differentially expressed transcripts related to metabolic processes in ovule under heat stress conditions.

| **Transcript Id** | **Log_2_ Fold Change** | **Transcript description** |
| --- | --- | --- |
| comp62491_c0_seq1 | -5.59 | probable 6-phosphogluconolactonase 2 |
| comp70287_c0_seq2 | -5.44 | aspartic proteinase |
| comp70903_c0_seq5 | -5.44 | alcohol dehydrogenase 1 |
| comp80307_c1_seq18 | -5.33 | BRASSINOSTEROID INSENSITIVE 1-associated receptor kinase 1 precursor |
| comp67676_c0_seq2 | -4.92 | asparaginyl endopeptidase REP-2 |
| comp67676_c0_seq2 | -4.92 | asparaginyl endopeptidase REP-2 |
| comp82638_c0_seq1 | -4.60 | transcription factor bHLH95 |
| comp84118_c0_seq2 | -4.81 | ABC transporter D family member 1-like |
| comp68443_c1_seq1 | -4.41 | senescence-associated protein, putative |
| comp73450_c0_seq1 | -4.37 | serine carboxypeptidase 1 |
| comp88236_c0_seq1 | -4.28 | polyphenol oxidase |
| comp88268_c0_seq1 | -4.27 | polyphenol oxidase |
| comp83060_c3_seq3 | -4.20 | glyceraldehyde-3-phosphate dehydrogenase |
| comp79153_c0_seq35 | -4.29 | beta-carotene hydroxylase 1 |
| comp86722_c0_seq16 | -4.17 | predicted protein |
| comp62499_c0_seq1 | -4.24 | triosephosphate isomerase, cytosolic |
| comp85492_c5_seq3 | -4.07 | S-adenosylmethionine decarboxylase proenzyme |
| comp59399_c1_seq1 | -4.22 | UDP-glycosyltransferase 91C1-like |
| comp85505_c0_seq1 | -4.19 | succinate dehydrogenase subunit 5, mitochondrial |
| comp73569_c0_seq3 | -4.04 | gibberellin 20 oxidase 2 |
| comp69688_c1_seq2 | -4.00 | thioredoxin H-type |
| comp80099_c0_seq3 | -4.19 | extradiol ring-cleavage dioxygenase-like |
| comp59399_c0_seq2 | -4.16 | UDP-glycosyltransferase 91C1-like |
| comp85809_c0_seq17 | -4.26 | protein ECERIFERUM 3-like |
| comp86095_c2_seq69 | -4.10 | NADH dehydrogenase subunit 7 |
| comp86139_c1_seq7 | -4.00 | beta-expansin EXPB7 |
| comp83923_c2_seq9 | -3.81 | histone H4 |
| comp73511_c0_seq1 | -3.72 | serine carboxypeptidase 1 |
| comp80099_c1_seq1 | -3.84 | extradiol ring-cleavage dioxygenase-like |
| comp83465_c0_seq4 | -3.74 | ATP synthase subunit gamma, mitochondrial |
| comp87305_c1_seq1 | -3.64 | ATPase subunit 1 (mitochondrion) |
| comp85721_c0_seq3 | -3.85 | T-complex protein 1 subunit epsilon |
| comp36884_c0_seq1 | -3.50 | adenine nucleotide transporter BT1, chloroplastic/amyloplastic/mitochondrial-like |
| comp86012_c3_seq2 | -3.83 | glyceraldehyde-3-phosphate dehydrogenase |
| comp74270_c0_seq5 | -3.46 | ascorbate peroxidase |
| comp80278_c1_seq6 | -3.59 | GDSL esterase/lipase At5g55050-like |
| comp79836_c1_seq5 | -3.69 | GTP-binding protein YPTM2 |
| comp78929_c0_seq1 | -3.49 | dihydrolipoyl dehydrogenase 1, chloroplastic-like |
| comp87485_c0_seq1 | -3.37 | signal anchor, putative |
| comp47450_c0_seq1 | -3.35 | probable apyrase 3 |
| comp84861_c0_seq5 | -3.39 | predicted protein |
| comp84313_c0_seq27 | -3.29 | ADP-glucose pyrophosphorylase endosperm large subunit |
| comp85732_c0_seq4 | -3.59 | ATPase subunit 4 (mitochondrion) |
| comp84200_c0_seq6 | -3.31 | carbamoyl-phosphate synthase small chain |
| comp81090_c3_seq4 | -3.14 | probable NADH dehydrogenase [ubiquinone] 1 alpha subcomplex subunit 12 |

**Supplementary Table S13** Details of up and down-regulated differentially expressed transcripts related to hormone biosynthesis in leaf under heat stress conditions.

| **Transcript Id** | **Log_2_ Fold Change** | **Transcript description** |
| --- | --- | --- |
| comp83741_c1_seq17 | 5.22 | ethylene-responsive transcription factor 3 |
| comp87982_c0_seq1 | 7.25 | undecaprenyl pyrophosphate synthetase |
| comp31579_c0_seq2 | 6.73 | terpene synthase 7 |
| comp85923_c1_seq18 | 6.53 | ABA 8'-hydroxylase |
| comp65718_c0_seq1 | 6.34 | alpha-dioxygenase 1-like |
| comp73090_c0_seq10 | 6.17 | ZIM motif family protein |
| comp77362_c1_seq2 | 5.74 | catalase |
| comp65718_c1_seq1 | 5.62 | alpha-dioxygenase 1-like |
| comp83408_c0_seq1 | 5.04 | zeaxanthin 7,8(7',8')-cleavage dioxygenase, chromoplast |
| comp21104_c0_seq1 | 7.49 | terpene synthase 7 |
| comp35072_c0_seq1 | 7.37 | undecaprenyl pyrophosphate synthetase |
| comp77346_c1_seq8 | 8.27 | dehydrin COR410 |
| comp81007_c0_seq1 | -5.71 | lipoxygenase |
| comp71785_c0_seq4 | -5.47 | IAA15 - auxin-responsive Aux/IAA family member |
| comp83970_c0_seq2 | -5.21 | reduced height-2 |
| comp77346_c0_seq9 | -5.11 | dehydrin COR410 |
| comp78895_c0_seq3 | -5.10 | lipoxygenase |
| comp77212_c1_seq51 | -5.06 | TPA: putative tify domain/CCT motif transcription factor family protein |
| comp83810_c1_seq3 | -4.51 | IAA-amino acid hydrolase ILR1-like 6 |
| comp81007_c0_seq10 | -3.93 | TPA: lipoxygenase1 |
| comp64321_c0_seq1 | -3.88 | calmodulin-like protein 4 |
| comp71072_c0_seq3 | -3.87 | Remorin DNA binding protein |
| comp64321_c1_seq1 | -3.84 | calmodulin-like protein 4 |
| comp39559_c0_seq1 | -3.81 | low temprature induced-like protein |
| comp84312_c0_seq4 | -3.76 | serine decarboxylase 1 |
| comp82490_c0_seq5 | -3.69 | malate dehydrogenase isoform 1 isoform X1 |
| comp79443_c0_seq5 | -3.66 | protein TIFY 10B-like |
| comp72252_c0_seq5 | -3.63 | CBL-interacting protein kinase 6-like |
| comp79443_c0_seq1 | -3.53 | protein TIFY 10B-like |
| comp85923_c1_seq20 | -3.50 | abscisic acid 8'-hydroxylase 3-like |
| comp77592_c0_seq2 | -3.43 | 4-hydroxy-3-methylbut-2-en-1-yl diphosphate synthase, chloroplastic |
| comp70082_c0_seq1 | -3.33 | GEM-like protein 5 |

**Supplementary Table S14** Details of up and down-regulated differentially expressed transcripts related to hormone biosynthesis in pollen under heat stress conditions.

| **Transcript Id** | **Log_2_ Fold Change** | **Transcript description** |
| --- | --- | --- |
| comp77758_c1_seq2 | 8.26 | late embryogenesis abundant protein Lea5-A-like |
| comp80320_c0_seq6 | 5.85 | calcium-dependent protein kinase 34-like |
| comp83792_c0_seq27 | 5.85 | putative calcium-dependent protein kinase |
| comp80320_c0_seq30 | 6.63 | calcium-dependent protein kinase 34-like |
| comp85186_c0_seq7 | -5.40 | zeaxanthin epoxidase |
| comp85030_c0_seq2 | -5.06 | probable indole-3-acetic acid-amido synthetase GH3.8 |
| comp80320_c0_seq5 | -4.69 | calcium-dependent protein kinase 34-like |
| comp72278_c0_seq2 | -4.18 | ZIM motif family protein |
| comp82123_c1_seq6 | -4.15 | gibberellin 2-oxidase |
| comp12375_c0_seq1 | -3.71 | phospho-2-dehydro-3-deoxyheptonate aldolase 1, chloroplastic-like |
| comp80446_c0_seq7 | -3.33 | predicted protein |

**Supplementary Table S15** Details of up and down-regulated differentially expressed transcripts related to hormone biosynthesis in ovule under heat stress conditions.

| **Transcript Id** | **Log_2_ Fold Change** | **Transcript description** |
| --- | --- | --- |
| comp87541_c0_seq1 | 6.64 | late embryogenesis abundant protein |
| comp59713_c0_seq1 | 6.60 | Early-methionine-labelled polypeptide |
| comp87560_c0_seq1 | 6.22 | late embryogenesis abundant protein |
| comp87533_c0_seq1 | 5.34 | seed maturation protein |
| comp81629_c0_seq26 | 4.87 | callose synthase 10 |
| comp87869_c0_seq1 | 4.72 | 18 kDa seed maturation protein-like |
| comp77362_c1_seq2 | 4.54 | catalase |
| comp84014_c0_seq3 | 4.46 | reduced height-2 |
| comp12406_c0_seq1 | 4.42 | RAB17 protein |
| comp87870_c0_seq1 | 4.32 | 18 kDa seed maturation protein-like |
| comp63308_c0_seq1 | 4.10 | uncharacterized membrane protein At3g27390 |
| comp62644_c0_seq3 | 4.01 | low temprature induced-like protein |
| comp62464_c0_seq3 | 3.92 | low temprature induced-like protein |
| comp85282_c0_seq19 | 3.85 | auxin response factor 4 |
| comp82242_c0_seq1 | 3.77 | oleosin 18 kDa |
| comp67757_c0_seq1 | 3.56 | oleosin 18 kDa |
| comp81770_c1_seq2 | 3.49 | low-temperature-induced 65 kDa protein |
| comp81921_c0_seq20 | 3.39 | protein IWS1 homolog isoform X1 |
| comp86501_c0_seq4 | 3.21 | phenylalanine ammonia-lyase |
| comp79158_c0_seq1 | -4.21 | Catalase-rel |

**Supplementary Table S16** List of Real-time PCR primers used for validation in the present study.

| **Selected leaf DEG for validation** | **Primer name** | **Primer sequence** |
| --- | --- | --- |
| comp86530_c0_seq12 | qZmHS1-F | 5ˊ-CGAGATCATTGCCAACGACC-3ˊ |
|  | qZmHS1-R | 5ˊ-AGACGCTTGGCATCAAAGAC-3ˊ |
| comp74270_c0_seq1 | qZmHS4-F | 5ˊ-AACGAACAAAGGGTCCCAAA-3ˊ |
|  | qZmHS4-R | 5ˊ-ACATTCTGCTGCCTGTATGG-3ˊ |
| comp85370_c0_seq1 | qZmHS23-F | 5ˊ-GGCTACGTGTTGAAGTCTGT-3ˊ |
|  | qZmHS23-R | 5ˊ-GTTTGCGACAGCAAGTTGAA-3ˊ |
| comp87665_c0_seq1 | qZmHS27-F | 5ˊ-CACTACAGCACGTACGGAAT-3ˊ |
|  | qZmHS27-R | 5ˊ-TTCGAGATCCTCGCCTTCTT-3ˊ |
| comp86585_c1_seq3 | qZmHS29-F | 5ˊ-CTCCGTCATTTGGTACAGCA-3ˊ |
|  | qZmHS29-R | 5ˊ-TTCCCTGCATATGACAGCAC-3ˊ |
| comp72022_c0_seq2 | qZmHS35-F | 5ˊ-CAAGGCCAAGGGAAAGAAGA-3ˊ |
|  | qZmHS35-R | 5ˊ-TAACCACGCTCATACCCTCT-3ˊ |
| 18S ribosomal RNA | 18S-F | 5ˊ-TGTGAAACTGCGAATGGCTCATTAA-3ˊ |
|  | 18S-R | 5ˊ-GAAGTCGGGATTTGTTGCACGTATT-3ˊ |

**Supplementary Table S17** Comparative analysis of Log_2_ fold value of qRT-PCR and transcriptome data.

| **S. No.** | **Candidate gene** | **LM 11** | **CML 25** | **ΔΔct** | **log2 (2-ΔΔct)** | **Transcriptome data** | **Function** |
| --- | --- | --- | --- | --- | --- | --- | --- |
| 1 | comp86530_c0_seq12 | 27.91 | 31.99 | 0.0591 | -4.08 | -4.2 | HSP70 |
| 2 | comp74270_c0_seq1 | 36 | 28.89 | 138.14 | 7.1 | 7.12 | peroxidase |
| 3 | comp85370_c0_seq1 | 30.8 | 31.36 | 0.1088 | -3.2 | -5.2 | tRNASAD |
| 4 | comp87665_c0_seq1 | 30.93 | 32.89 | 0.1947 | -2.3 | -4.9 | Steroiddh |
| 5 | comp86585_c1_seq3 | 34.26 | 33.21 | 0.1948 | -2.3 | -3.4 | Pectinesterase |
| 6 | comp72022_c0_seq2 | 28.45 | 31.23 | 0.1560 | -2.6 | -3.7 | Mitochondrial matrix |
